# Pharmacokinetic interaction assessment of an HIV broadly neutralizing monoclonal antibody VRC07-523LS: a cross-protocol analysis of three phase 1 trials in people without HIV

**DOI:** 10.1186/s12865-025-00687-7

**Published:** 2025-02-19

**Authors:** Tariro D. Chawana, Stephen R. Walsh, Lynda Stranix-Chibanda, Zvavahera M. Chirenje, Chenchen Yu, Lily Zhang, Kelly E. Seaton, Jack Heptinstall, Lu Zhang, Carmen A. Paez, Theresa Gamble, Shelly T. Karuna, Philip Andrew, Brett Hanscom, Magdalena E. Sobieszczyk, Srilatha Edupuganti, Cynthia L. Gay, Sharon B. Mannheimer, Christopher B. Hurt, Kathryn E. Stephenson, Laura L. Polakowski, Hans Spiegel, Margaret Yacovone, Stephanie Regenold, Catherine Yen, Jane AG. Baumblatt, Lucio Gama, Dan H. Barouch, Estelle Piwowar-Manning, Richard A. Koup, Georgia D. Tomaras, Ollivier Hyrien, Alison C. Roxby, Yunda Huang, on behalf of the HVTN 127/HPTN 087, HVTN 130/HPTN 089 and HVTN 136/HPTN 092 Study Teams

**Affiliations:** 1 University of Zimbabwe Clinical Trials Research Centre (UZ-CTRC), Harare, Zimbabwe; 2 Harvard Medical School, Boston, MA USA; 3 Bixby Centre for Global Reproductive Health, University of California San Francisco, San Francisco, USA; 4 Vaccine and Infectious Disease Division, Fred Hutchinson Cancer Centre, Seattle, WA USA; 5 Duke University, Durham, NC USA; 6 FHI 360, Durham, NC USA; 7 Columbia University, New York, NY, Columbia USA; 8 Emory University School of Medicine, Atlanta, USA; 9 Institute for Global Health and Infectious Diseases, University of North Carolina at Chapel Hill, Chapel Hill, NC USA; 10 Division of AIDS, National Institute of Allergy and Infectious Diseases, Bethesda, MD USA; 11 Vaccine Research Centre, National Institute of Allergy and Infectious Diseases, Bethesda, MD USA; 12 Center for Virology and Vaccine Research, Beth Israel Deaconess Medical Center, Boston, MA USA; 13 School of Medicine, Department of Pathology, Johns Hopkins University, Baltimore, MD USA

**Keywords:** Monoclonal antibodies, VRC07-523LS, Combination administration, Single administration, HIV prevention, Pharmacokinetics

## Abstract

VRC07-523LS is a safe and well-tolerated monoclonal antibody (mAb) targeting the CD4 binding site on the HIV envelope (Env) trimer. Efficacy of VRC07-523LS, in combination with mAbs targeting other HIV epitopes, will be evaluated in upcoming trials to prevent HIV acquisition in adults. However, differences in the pharmacokinetics (PK) of VRC07-523LS when administered alone vs. in combination with other mAbs have not been formally assessed. We performed a cross-protocol analysis of three clinical trials and included data from a total of 146 adults without HIV who received intravenous (*n* = 95) or subcutaneous (*n* = 51) VRC07-523LS, either alone (‘single’; *n* = 100) or in combination with 1 or 2 other mAbs (‘combined’; *n* = 46). We used an open, two-compartment population PK model to describe serum concentrations of VRC07-523LS over time, accounting for inter-individual variabilities. We compared individual-level PK parameters between the combined vs. single groups using the targeted maximum likelihood estimation method to adjust for participant characteristics. No significant differences were observed in clearance rate, inter-compartmental clearance, distribution half-life, or total VRC07-523LS exposure over time. However, for the combined group, mean central volume of distribution, peripheral volume of distribution, and elimination half-life were slightly greater, corresponding to slightly lower predicted concentrations early post-administration with high levels being maintained in both groups. These results suggest potential PK interactions between VRC07-523LS and other mAbs, but with small clinical impact in the context of HIV prevention. Our findings support coadministration of VRC07-523LS with other mAbs, and the use of the developed PK models to design future trials for HIV prevention.

**Supplementary Information:**

The online version contains supplementary material available at 10.1186/s12865-025-00687-7.

## Introduction

In the past decades, antiretroviral therapy (ART), pre- and post-exposure prophylaxis (PrEP and PEP, respectively), condom use, counselling and other interventions have reduced HIV-related morbidity and mortality, and HIV acquisition. However, approximately 1.3 million people acquired HIV in 2023, adding to the 39 million people who are living with HIV [1]. Africa remains the region most affected, with incident HIV diagnoses disproportionately high among adolescent girls and young women [1–5], suggesting new and more widely accessible approaches for HIV prevention are needed.

The use of PrEP is an established approach to preventing new HIV acquisition [6]. Oral emtricitabine/tenofovir diphosphate (TDF)-based PrEP is affordable and highly efficacious, but dependent on an individual’s continuing daily or event-driven dosing as prescribed [7–10]. Long-acting cabotegravir injection every 8 weeks is superior to and has greater acceptability than oral PrEP, but this method remains prohibitively expensive and inaccessible [8, 11–17]. Additionally, long-acting lenacapavir given twice yearly demonstrated excellent (almost zero incident acquisitions in the study arm during trials) and superiority to oral PrEP, but isn’t yet accessible and may be expensive [18]. An effective HIV vaccine remains elusive, and recent trials (HVTN 702/Uhambo, HVTN 705/Imbokodo, HVTN 706/Mosaico and PrEPVacc) have not shown efficacy [19–21].

There is more than 100 years of experience with using antibodies as PrEP (e.g., measles, polio, cytomegalovirus, hepatitis A, and respiratory syncytial virus infections), PEP (e.g., hepatitis B, rabies and varicella zoster infections) and treatment (e.g., SARS-CoV-2 infection) [2, 22–37]. This long history also adds weight to the hypothesis that mAbs could successfully prevent HIV infection. Passive immunization with broadly neutralizing monoclonal antibodies (mAbs) has shown promise as an additional option for HIV prevention due to less dependance on individual adherence and other motivations (e.g., perception as a "natural" intervention, positive attitudes toward intravenous drips or infusions, infrequent administration, etc.) [2, 38]. Bioengineered immunoglobulin G (IgG-backboned) mAbs with the LS-mutation (M428L/N434S) in the constant fragment crystallizable (Fc) region may be administered every 6 months given their extended half-lives [39, 40].

Two proof-of-concept Antibody Mediated Prevention (AMP) trials showed that, at sufficient concentrations [41], a single mAb (VRC01) could prevent infection against viral strains highly sensitive to neutralization in vitro [39, 42]. However, a single-antibody approach for HIV, a virus with high replication, high mutation rates, and globally diverse circulating clades, is likely limited due to viral diversity, viral escape, and potential for development of resistance to any single mAb. To address these concerns, studies of combinations of mAbs targeting different epitopes, or bi- or tri-specific mAbs to increase breadth and potency, and to limit viral resistance are currently underway [2, 39, 43]. Multiple early-phase trials have confirmed that mAbs are safe and well-tolerated when administered alone or in combination [32, 33, 44–48]. However, pharmacokinetic (PK) data on combinations of anti-HIV-1 mAbs are limited, and the impact of co-administering multiple HIV-1 mAbs on their individual PK has not been formally evaluated and remains a key unknown for designing effective mAb combinations against HIV acquisition.

When multiple IgG mAbs are co-administered, they may compete for Fc receptor binding and the neonatal fragment crystallizable receptor (FcRn) recycling pathways, potentially impacting their half-life and overall efficacy. Significant alterations in FcRn recycling efficiency are unlikely with doses less than around 10 mg/kg, which increase the total amount of IgG in the circulation by less than 1–2% [49]. On the other hand, at high concentrations of IgG, the FcRn recycling mechanism could have limited capacity. For example, FcRn can become saturated, leading to increased degradation and a shorter half-life for IgG due to a higher fractional catabolic rate. This phenomenon has been observed in high-dose intravenous immunoglobulin (IVIG) therapy, which significantly increases IgG clearance and reduces endogenous antibody concentrations [50, 51]. Co-administration of mAbs could impact the PK of individual mAbs by altering the rate of transfer between tissue and blood compartments, or the rate at which they are eliminated, possibly due to increasing competition for the receptors that make these transfer possible. The unique PK profiles of individual mAbs may present challenges in co-administration and delivery as regimens are designed to maintain the concentrations of all antibodies in the combination for optimal protective efficacy [52].

VRC07-523LS, an engineered variant of a clonal relative of VRC01, targets the CD4 binding site on the HIV envelope trimer. It has been shown to be generally safe and well tolerated in phase 1 trials, including HIV Vaccine Trials Network (HVTN) 127/HIV Prevention Trials Network (HPTN) 087, HVTN 130/HPTN 089 and HVTN 136/HPTN 092 [40, 53, 54]. These studies have also demonstrated that VRC07-523LS has an improved elimination half-life and neutralization profile compared to VRC01 [55]. In combination with mAbs targeting other HIV epitopes, VRC07-523LS is a strong candidate for inclusion in antibody efficacy evaluations for HIV prevention [40, 56–61]. Individual phase 1 trials of VRC07-523LS enrolled small numbers of participants, limiting the ability within each trial to make conclusions about its PK when combined with other mAbs [40, 53]. Mayer *et al* conducted a cross-protocol analysis of 16 clinical trials, including combination and/or single mAb regimens, with a goal of assessing whether the LS-mutation could improve PK profiles of mAbs compared to parental mAbs [58]. However, no direct comparisons of PK between single vs. combination administration were reported. Here, we describe the results of a cross-protocol analysis of the three phase 1 trials to assess potential PK interactions of VRC07-523LS when administered in combination with 1 or 2 other mAbs. We hypothesized that the overall PK of VRC07-523LS would be similar when administered in combination versus alone.

## Materials and methods

### Participants and study design

**Fig. 1:**
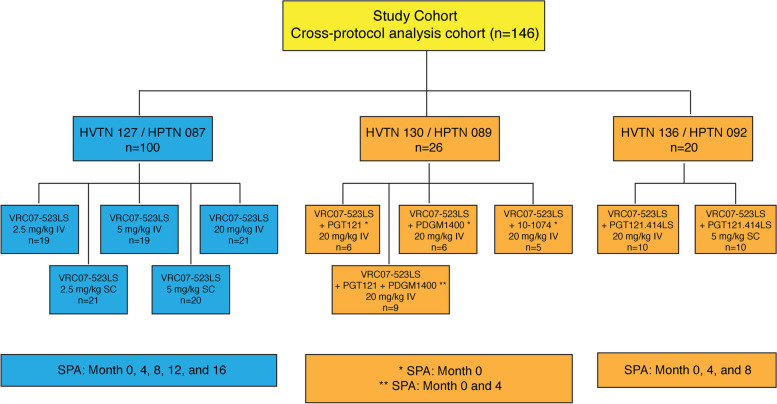
Description of participants, study regimens, and study products included in the cross-protocol analysis of three studies. Blue boxes represent single VRC07-523LS administration regimens, orange boxes represent regimens with VRC07-523LS administered in combination with one or two other mAbs. The combination group received the mAbs sequentially, with VRC07-523LS being given last in all cases. *Indicates administration of the mAbs once at month 0. **Indicates administration of the mAbs twice: at month 0 and at month 4. Abbreviations: IV- intravenous; SC- subcutaneous, SPA-study product administration

This was a retrospective, cross-protocol analysis of HVTN 127/HPTN 087 (NCT03387150), HVTN 130/HPTN 089 (NCT03928821) and HVTN 136/HPTN 092 (NCT04212091): three phase 1, randomized, multicenter trials conducted by the HVTN and HPTN (Figure 1) [40, 53, 62]. Participants aged 18–50 years without HIV were recruited from the United States (US) in all 3 trials and additionally from Switzerland in HVTN 127/HPTN 087. Primary outcomes of safety, tolerability, PK, and in vitro antiviral activity were evaluated for five antibodies or combinations thereof across the three trials: VRC07-523LS alone (HVTN 127/HPTN 087); VRC07-523LS combined with parental mAbs V3-glycan-targeting PGT121, V1/V2-glycan-targeting PGDM1400, or V3-glycan-targeting 10–1074 (HVTN 130/HPTN 089); and VRC07-523LS combined with LS-mAb V3-glycan-targeting PGT121.414.LS (HVTN 136/HPTN 092). Data from participants who received VRC07-523LS via the intravenous or subcutaneous route were included in this analysis. Participants in this analysis were pooled into two groups based on whether they received VRC07-523LS with one or two other mAbs (‘combination’ group) or received VRC07-523LS alone (‘single’ group). The combination group received the mAbs sequentially, not combined in one administration.

### Procedures

Serum concentrations were measured 1, 3, 6, 14, 28, 56, and 84 days after the first administration, and every 4–8 weeks after later administrations. To avoid the influence of different sampling timepoints on the PK parameter estimates, we excluded the one-hour post-infusion PK data collected in HVTN 136/HPTN 092 from our analyses, as samples were not drawn at that time point in the two other studies. Serum concentrations of VRC07-523LS after intravenous or subcutaneous administration were quantified by validated binding antibody multiplex assay (BAMA) at a central HVTN laboratory in all three trials, and by validated enzyme linked immunosorbent assay (ELISA) at a single laboratory for samples collected up to the second study product administration in HVTN 127/HPTN 087 [58, 62]. The lower limit of quantification (LLoQ) for VRC07-523LS was 1.0 μg/ml by ELISA and 0.0457 μg/ml by BAMA [62, 63].

### Statistical and pharmacokinetic (PK) analysis

Two analysis approaches were used to compare the PK of VRC07-523LS between combination vs. single administrations. In Approach 1 (primary analysis), individual-level PK parameters were first estimated from a base population PK (popPK) model without any covariate adjustment. Estimated individual-level PK parameters and additional derived PK features were then compared between combination vs. single administrations via the targeted maximum likelihood estimation (TMLE) method to adjust for individual-level covariates that could differ between the two groups and/or are predictive of the PK parameters [64–69]. More details on the PK modeling and the TMLE method are provided in the next subsections. In Approach 2 (supportive analysis), a covariate (‘coadministration’) delineating combination vs. single administration was included in a covariate-adjusted popPK model to evaluate the influence of coadministration on various PK parameters. The same set of individual-level covariates included in Approach 1 were considered as potential covariates in Approach 2, and the final covariate-adjusted popPK model was selected through a model selection procedure as described in the next subsection.

One key advantage of Approach 1 is that it provides quantitative estimates and statistical inferences on the effect of coadministration (combination vs. single) on all PK features of interest from a causal framework that isolates the effect while minimizing bias and maximizing precision of the comparisons. On the other hand, Approach 2 is commonly considered in the field, although the interpretation of the effect of coadministration on PK parameters is conditional on the levels of other covariates included in the final model (i.e., not causal). In addition, the final model is determined by having the best model that fits the data and explains the variations, not by isolating the effect of coadministration. Another drawback of Approach 2 is that it is not directly feasible to assess the influence of coadministration on PK features that are not included in the parametrization of the final popPK model. For the above reasons, Approach 1 is considered the primary approach and Approach 2 supportive in our analyses. We considered adjusted *p*-values < 0.05 as significant.

#### popPK modeling

The individual-level PK of VRC07-523LS following intravenous and subcutaneous administration was described by an open two-compartment model with first-order elimination from the central compartment. Two-compartment models, which assume that antibody clearance is bi-phasic, have been previously used to describe the PK of multiple IgG mAbs, including VRC07-523LS [62]. The model was parameterized in terms of the central volume (Vc, L), clearance from the central compartment (CL, L/day), peripheral volume (Vp, L), and intercompartmental clearance (Q, L/day). For subcutaneous administrations, a depot compartment was added to characterize first-order absorption via the subcutaneous route with 2 parameters: bioavailability (F, %) and absorption rate (ka, day^−1^).

popPK models were used to account for inter-individual variabilities of PK parameters and residual errors. Non-linear mixed effects models were fitted with PK parameters estimated via the maximum likelihood method using the Stochastic Approximation Expectation Maximization (SAEM) algorithm as implemented in the Monolix software system (Version 2021 R2) [70]. We parameterized population-level (average) parameters as fixed effects and described inter-individual variation of these parameters by random effects and their correlations. We did not include random effects for absorption rate and bioavailability due to limited data availability to estimate these parameters. Residual error variance was modeled as the sum of 2 terms: one constant additive error term and another error term proportional to the conditional expectation of the concentration. Individual-level PK parameters were assumed to follow log-normal distributions.

In the base popPK model, no covariates were considered. To prepare for the subsequent TMLE analysis, using the base popPK model, we predicted participant-specific model parameters using their empirical Bayes estimates, defined as the most probable value given the estimated population parameters and the data from the given participant. We predicted these (random) individual-specific parameters by the mode of their conditional posterior distribution. They were subsequently used to predict the most probable trajectory of the serum concentration for the corresponding participant.

In the covariate-adjusted popPK model, besides coadministration (combination vs. single), body weight, creatinine clearance, age, and sex-at-birth were considered as potential covariates to explain variability of the PK parameters. For model selection, the COSSAC (COnditional Sampling use for Stepwise Approach based on Correlation tests) covariate model building algorithm was employed to select the final model [71].

#### Targeted maximum likelihood estimation (TMLE) method

Individual-level PK parameters estimated from the base popPK model without any covariate adjustment were compared between combination and single administrations using the TMLE method which adjusted for potential differences in participant baseline covariates. TMLE is an alternative to standard linear or non-linear regression and it leverages machine learning to improve robustness and efficiency, and reduce confounding bias, especially for comparisons between nonrandomized groups [64–69]. The average treatment effect (ATE) is a form of causal effects defined as the difference in expected VRC07-523LS (log-transformed) PK parameter values if everyone in the population received the combination regimen versus if everyone received the single regimen. The following potential confounders and predictors of PK variability that were previously identified [55, 72] were included: baseline body weight (kg), creatinine clearance (mL/min), age (years), and sex assigned at birth (female vs. male). ). Creatinine clearance was calculated using the Cockroft Gault equation with correction for female sex at birth (0.85) [73]. This analysis approach was carried out using the TMLE package (version 1.5.0.2) in R (version 4.2.1) [74].

All TMLE estimation results were averaged over 20 runs with a fixed random seed on top of the 10-fold cross validation estimation procedure to ensure stability of the estimates. The set of learning algorithms used by TMLE for estimating the mean PK feature and ATE conditional on baseline covariates included the following: SL.glm, SL.step, SL.ranger, SL.earth, SL.glmnet, and SL.mean as described previously [72]. In addition, a bootstrap procedure based on 500 sampled-with-replacement datasets was used to estimate the variance of the mean of various PK feature estimates for each of the combination or single groups and the mean group difference of the log-transformed PK parameters, derive the 95% confidence intervals (CIs), and test for a non-zero mean difference between combination and single administration groups. Bootstrap accounts for variability and co-variability of the individual level estimates for each PK feature since they were derived from a common popPK model. The Holm procedure was used to adjust the resulting *p*-values for comparisons of the multiple PK features between the combination and single administration groups [75].

## Results

### Participant cohort and characteristics

We analyzed data from a total of 146 participants who received VRC07-523LS via intravenous or subcutaneous administration, 100 of whom received VRC07-523LS alone (‘single’) and 46 received VRC07-523LS with one or two other mAbs (‘combination’) (Figure 1). Primary results from each parent study are described elsewhere [40, 53, 62]. From HVTN 127/HPTN 087, 100 of 124 participants were included in this analysis; twenty-one were excluded because they received VRC07-523LS via the intramuscular route and three were excluded because they were in the placebo arm. Intramuscular administration was only performed in a single study (HVTN 127/HPTN 087) and only with VRC07-523LS alone, never in combination with other bnAbs. Therefore, these data would not contribute meaningfully to the cross-protocol analysis. From HVTN 130/HPTN 089, 26 of 27 participants were included here; one was excluded because they did not receive VRC07-523LS due to infiltration of the intravenous site. From HVTN 136/HPTN 092, 20 of 32 participants were included; the remaining twelve did not receive VRC07-523LS.

Most participants (100% in the combination and 92% in the single groups) included in our analysis were from the US (Table 1). The mean age was 28 years across both the combination and single groups. There were slightly more people who identified as female at birth in the single group (61% vs. 52%), and this group had a slightly higher body weight (76 kg vs. 71 kg) at baseline compared to the combination group. Baseline creatinine clearance was comparable between the two groups. These factors, except country of study, were considered as potential covariates to adjust for in both Approach 1 and Approach 2 to compare PK feature differences between combination and single administrations of VRC07-523LS.

**Table 1:** Summary of baseline demographic characteristics

Characteristic	Combination: VRC07-523LS with 1 or 2 other HIV mAbs, *N* = 46	Single: VRC07-523LS alone, *N* = 100
**Sex assigned at birth**		
Female [n (%)]	24 (52%)	61 (61%)
Male [n (%)]	22 (48%)	39 (39%)
**Weight (kg) [median, (range)]**	71 (46, 109)	76 (48, 114)
**Creatinine clearance (mL/min) [median, (range)]**	120 (73, 183)	122 (66, 220)
**Age (years) [median, (range)]**	28 (19, 50)	28 (18, 50)
**Country of study**		
United States [n (%)]	46 (100%)	92 (92%)
Switzerland [n (%)]	0 (0%)	8 (8%)

### Base popPK modeling without covariate adjustment

**Fig. 2:**
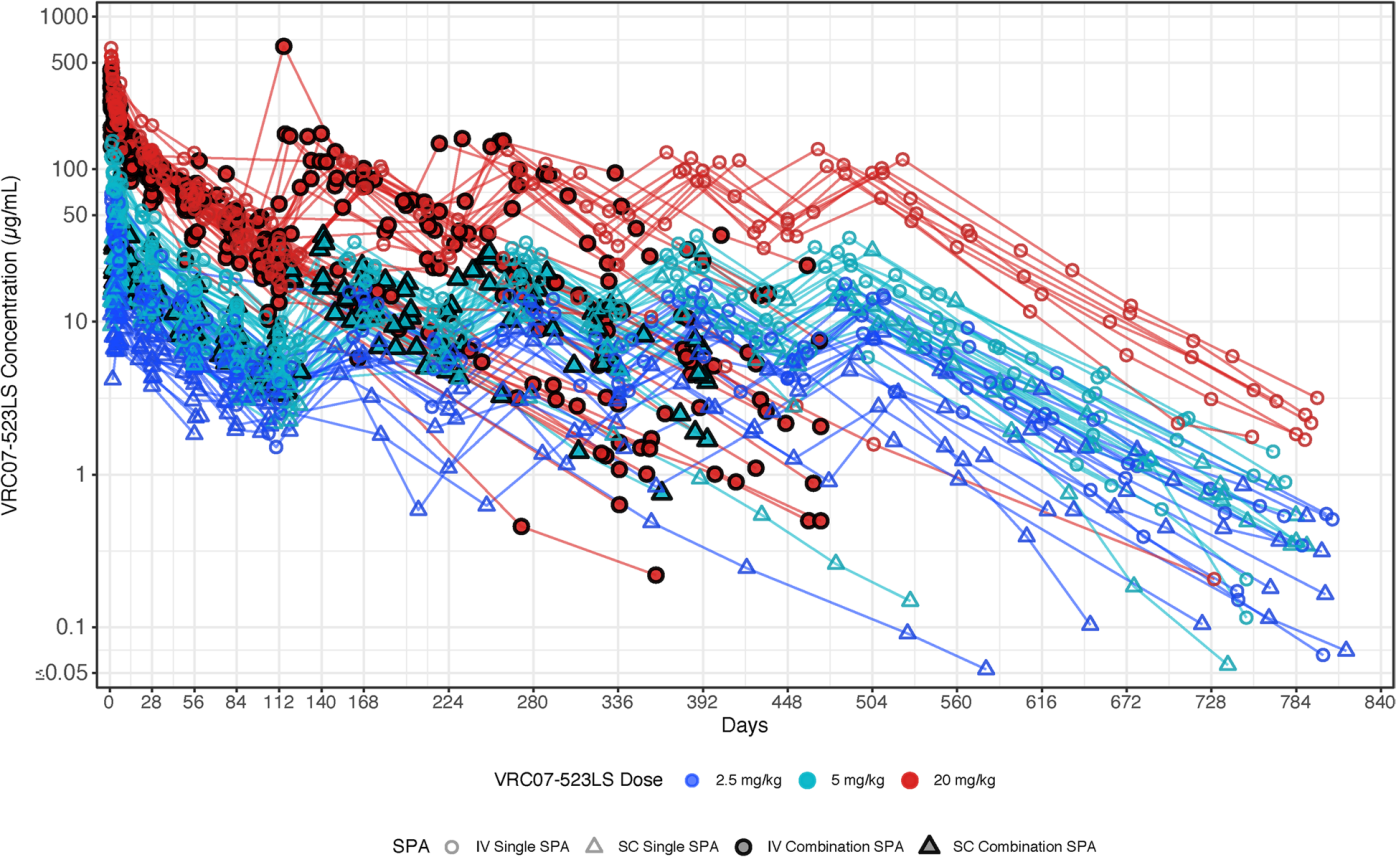
Observed individual-level VRC07-523LS serum concentrations over time. Colors represent different dose levels of VRC07-523LS at 2.5, 5 or 20 mg/kg; shapes represent route of administration via subcutaneous or intravenous; filled vs. open symbols represent combination vs. single administrations, respectively. Each line in the graph indicates a unique participant. Figure S2 shows observed individual-level VRC07-523LS serum concentrations over time delineated by intravenous and subcutaneous SPA. Figure S3 shows observed individual-level VRC07-523LS serum concentrations over time delineated by combination and single SPA. Abbreviations: IV- intravenous; SC- subcutaneous; SPA: study product administration

The observed, individual-level serum concentrations of VRC07-523LS over time are displayed in Figure 2 and Figures S1-S3. Overall, higher concentrations of VRC07-523LS were observed when higher doses were administered for each administration route, but consistent decay slopes were observed across dose levels. No differences in the observed concentrations were apparent between the combination vs. single groups. popPK models based on the pooled data were hence considered to characterize the overall patterns while accounting for between-individual variabilities in PK via the incorporation of random effects in the model. Based on the base popPK model without covariate adjustment, the estimated population-level subcutaneous (vs. intravenous) bioavailability was 0.47 (47%), clearance was 0.12 L/day, volume of central compartment was 3.88 L, volume of peripheral compartment was 3.60 L and intercompartmental clearance was 0.30 L/day (Table 2).

**Table 2:** Estimates of PK parameters of VRC07-523LS from a base two-compartment popPK model

Parameter	Description	Estimate	95% CI	% RSE
Fixed Effects				
*F*	Bioavailability	0.47	(0.45, 0.49)	2.50
*Ka (/day)*	Absorption rate constant	0.34	(0.27, 0.43)	11.75
*CL (L/day)*	Clearance rate	0.12	(0.11, 0.13)	2.61
*Vc (L)*	Central volume	3.88	(3.56, 4.23)	4.36
*Q (L/day)*	Inter-compartmental clearance	0.30	(0.26, 0.35)	7.83
*Vp (L)*	Peripheral volume	3.60	(3.34, 3.89)	3.85
Random Effects				
*ωCL*	SD, clearance	0.29	(0.25, 0.33)	6.52
*ωVc*	SD, central volume	0.40	(0.34, 0.46)	7.44
*ωQ*	SD, inter-compartmental clearance	0.57	(0.45, 0.73)	12.65
*ωVp*	SD, peripheral volume	0.24	(0.19, 0.29)	10.57
Error Model Parameters				
σ1	SE, additive error	0.13	(0.09, 0.19)	20.39
σ2	SE, proportional error	0.14	(0.13, 0.15)	3.45

The model included serum concentrations from 146 participants who received intravenous or subcutaneous VRC07-523LS alone (*n* = 46) or in combination with other mAbs (*n* = 100) across three phase 1 clinical trials. No covariates were adjusted for in the base popPK model

*Abbreviations*: *CI* confidence interval, *%RSE* % relative standard error, *SD* standard deviation, *SE* standard error

**Fig. 3:**
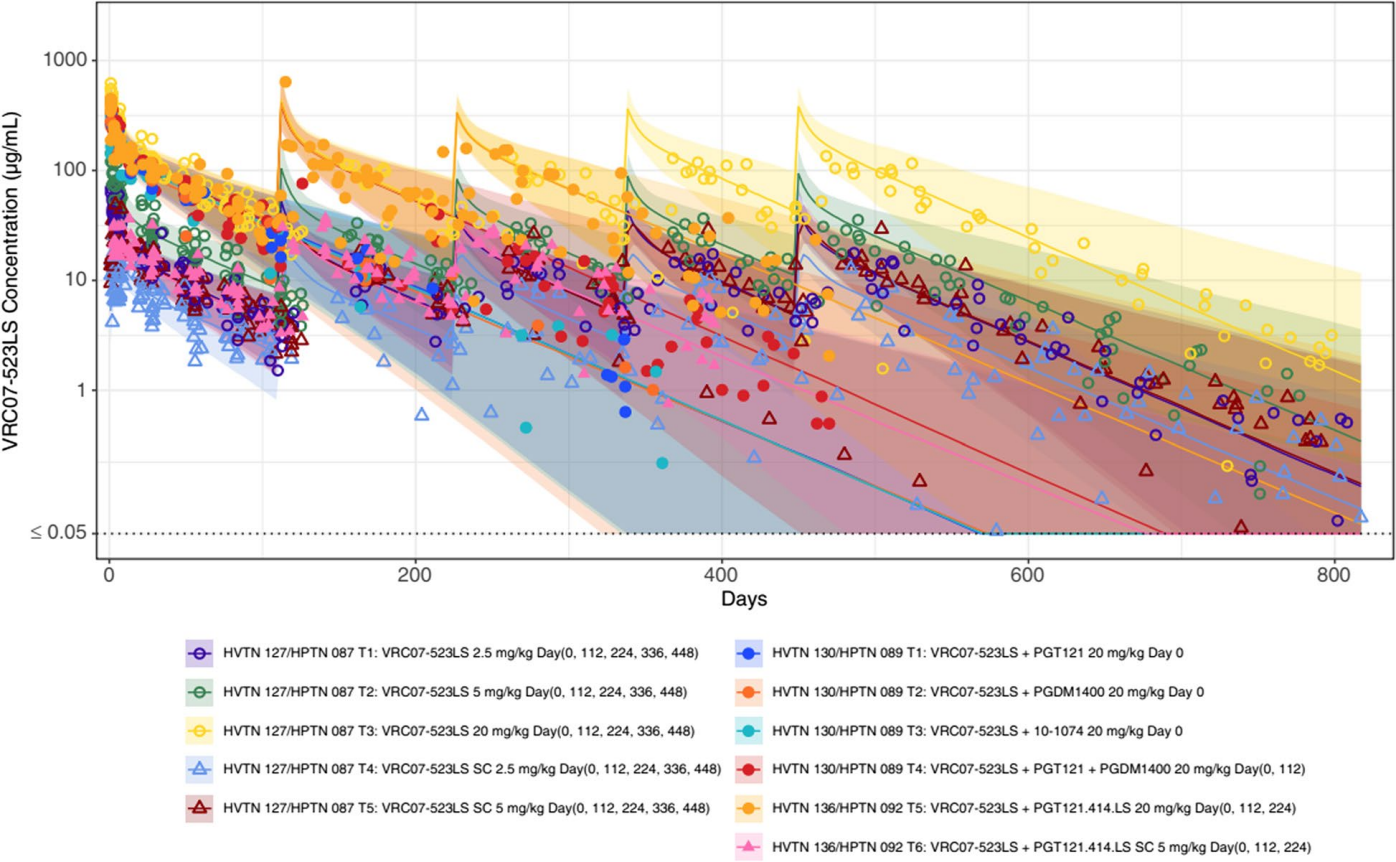
Observed VRC07-523LS concentrations with 90% prediction interval from the base popPK model. Symbols and colors represent the observed concentrations from different dosing regimens with circles indicating those after intravenous administration and triangles indicating those after subcutaneous administration. Solid lines represent the predicted median concentration of each study regimen, with shaded areas denoting the 90% prediction interval

Overall, the base popPK model fit the data well with most of the observed concentrations falling within the 90% prediction intervals (Fig. 3). When the model-based individual-level predicted concentrations were plotted against the observed concentrations, there was a general agreement, indicating a good model fit (Figure S4). This formed the basis for the subsequent comparison of the individual-level PK features between the combination and single groups. The individual-level PK parameter estimates are summarized by combination and single administration in Table S1.

**Fig. 4:**
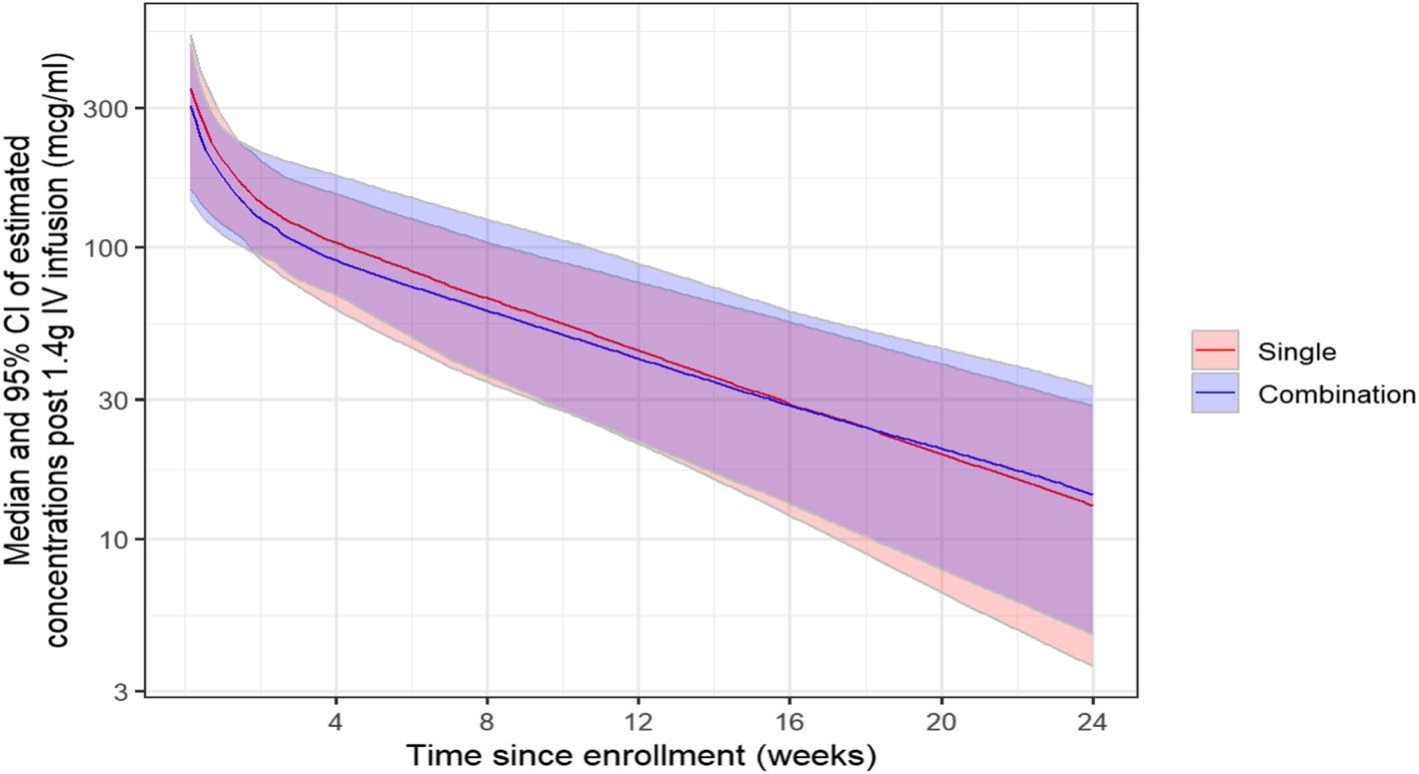
Estimated VRC07-523LS serum concentrations over time via the base popPK model. Red line and purple shaded area represent the median and 95% empirical confidence interval, respectively, of simulated concentrations after intravenous administration of VRC07-523LS alone at 1.4 g. Blue line and blue shaded area represent the median and 95% empirical confidence interval, respectively, of simulated concentrations after an IV administration of VRC07-523LS at 1.4 g co-administered with other mAbs. PK parameter estimate values from the base popPK model were used in these simulations, with the dose level of 1.4g being equivalent to 20 mg/kg for a 70kg person

To visually illustrate VRC07-523LS PK patterns between the two administration groups, estimated individual-level PK parameters from the base popPK model were used to simulate VRC07-523LS concentrations continuously (daily grid) after intravenous administration of VRC07-523LS for the single and combination group participants. As shown in Fig. 4, concentrations were initially lower in the combination group until about 16 weeks post intravenous administration. At approximately 16 weeks, combination and single concentrations were largely overlapping due to a slower decay in the combination group. After around 18 weeks, concentrations were higher in the combination group, leading to an overall simillar drug exposure (i.e., area under the curve) between the combination and single administration groups.

### Comparisons of VRC07-523LS PK features via TMLE (Approach 1)

**Fig. 5:**
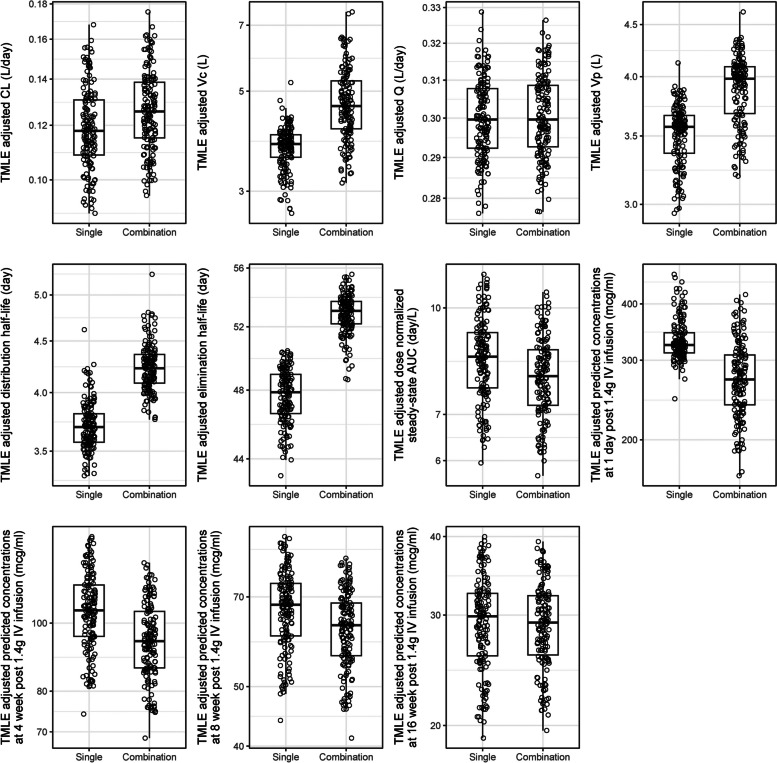
Distributions of covariate-adjusted individual-level PK features via the targeted maximum likelihood estimation (TMLE) method (Approach 1). Each dot indicates the TMLE-adjusted PK feature estimate for each participant as if all 146 received VRC07-523LS alone (left box), or all 146 received VRC07-523LS in combination with other mAbs (right box) via TMLE. The mid-line of the box denotes the median and the ends of the box denote the 25th and 75th percentiles. The whiskers at the top and bottom of the box extend to the most extreme data points that are no more than 1.5 times the interquartile range (i.e., height of the box) or if no value meets this criterion, to the data extremes. Abbreviations: TMLE- Targeted maximum likelihood estimation; CL- clearance rate; Vc- central volume; Q- inter-compartmental clearance; Vp- peripheral volume; AUC- area under the curve; IV- intravenous

In Approach 1, we extracted the estimated PK features of VRC07-523LS, including various PK parameters and concentrations at multiple post-administration days, from the base popPK model, and compared them between combination and single administrations. These estimated PK features prior to covariate adjustment are displayed for the two administrations in Figure S5. These estimated PK features after adjusting for body weight, creatinine clearance, age and sex-at-birth via TMLE are displayed by administration type as if all 146 participants received VRC07-523LS alone (single) or all received VRC07-523LS in combination with other mAbs (combination) in Figure 5. Specifically, as shown in Table 3, for the various PK parameters we found that the mean covariate-adjusted Vc was 25% larger (4.66 vs. 3.74 L, adjusted-*p*<0.001), Vp was 11% higher (3.89 vs. 3.51 L, adjusted-*p*=0.01), and elimination half-life was 11% longer (53 vs. 48 days, adjusted *p*=<0.01) for combination versus single administration. There were no significant differences in VRC07-523LS CL (0.13 L/day vs. 0.12 L/day, adjusted *p*=0.17), Q (0.3 L/day vs. 0.3 L/day, adjusted-*p*=0.97), distribution half-life (4.23 days vs. 3.71 days, adjusted *p*=0.06) or area under the concentration curve (AUC) (7.94 days/L vs. 8.42 days/L, adjusted *p*=0.17). For predicted concentrations of VRC07-523LS after intravenous administration at 1.4 g (or 20 mg/kg for a 70 kg person), we found that the predicted mean concentrations were initially lower at 1 day (271.34 vs. 332.68 mcg/ml, adjusted *p*<0.001) and 4 weeks (94.28 mcg/ml vs. 103.99 mcg/ml, adjusted *p*=<0.01) for combination versus single administration. In contrast, predicted mean concentrations were not different at 8 weeks (61.75 m vs. 66.73 mcg/ml, adjusted *p*=0.09) and 16-week (28.86 vs. 29.06 mcg/ml, adjusted *p*=1.00) between the two administrations.

**Table 3:** Comparisons of covariate-adjusted mean values of PK features of VRC07-523LS between single and combination administrations via the TMLE method

PK features	Description	Combination	Single	Ratio: Combination vs. Single	Unadjusted *p*-value	Adjusted *p*-value
		Mean (95% CI)				
**CL (L/day)**	Clearance from the central compartment	0.13 (0.12, 0.13)	0.12 (0.11, 0.13)	1.06 (1.00, 1.13)	0.06	0.22
**Vc (L)**	Volume of the central compartment	4.66 (4.07, 5.34)	3.74 (3.26, 4.29)	1.25 (1.14, 1.37)	< 0.001	< 0.001
**Q (L/day)**	Inter-compartmental distribution clearance	0.30 (0.22, 0.41)	0.30 (0.22, 0.41)	1.00 (0.91, 1.11)	0.97	1.00
**Vp (L)**	Volume of the peripheral compartment	3.89 (3.45, 4.38)	3.51 (3.07, 4.01)	1.11 (1.04, 1.18)	< 0.001	0.007
**Distribution half-life (day)**	Length of time for serum concentration of the mAb to decrease by half in the distribution phase	4.23 (3.15, 5.69)	3.71 (2.75, 5.01)	1.14 (1.03, 1.27)	0.01	0.086
**Elimination half-life (day)**	Length of time for serum concentration of the mAb to decrease by half in the elimination phase	52.84 (50.17, 55.66)	47.67 (45.46, 49.98)	1.11 (1.05, 1.17)	< 0.001	0.002
**Dose normalized steady-state AUC (day/L)**	Dose-normalized area under the curve in steady state after IV administration of 1.4 g	7.94 (7.52, 8.39)	8.42 (7.96, 8.9)	0.94 (0.89, 1)	0.06	0.224
**Predicted concentrations at 1 day (mcg/ml)**	Post 1.4 g IV infusion	271.34 (244.02, 301.71)	332.68 (299.8, 369.17)	0.82 (0.75, 0.88)	< 0.001	< 0.001
**Predicted concentrations at 4-week (mcg/ml)**	Post 1.4 g IV infusion	94.28 (89.34, 99.49)	103.99 (98.15, 110.18)	0.91 (0.86, 0.96)	< 0.001	0.004
**Predicted concentrations at 8-week (mcg/ml)**	Post 1.4 g IV infusion	61.75 (58.02, 65.71)	66.73 (62.81, 70.89)	0.93 (0.87, 0.99)	0.02	0.091
**Predicted concentrations at 16-week (mcg/ml)**	Post 1.4 g IV infusion	28.86 (26.78, 31.11)	29.06 (26.96, 31.33)	0.99 (0.91, 1.08)	0.88	1.00

*Abbreviations*: *CI* confidence interval, *CL* clearance rate, *Vc* central volume, *Q* inter-compartmental clearance, *Vp* peripheral volume, *mAb* monoclonal antibody, *AUC* area under the curve, *IV* intravenous

### Comparisons of VRC07-523LS PK features via covariate-adjusted popPK model (Approach 2)

In Approach 2, the final covariate-adjusted popPK model included the effect of coadministration (combination vs. single) on Vp and Vc, as well as the effect of body weight on CL, Q and Vp (Table S2). Consistent with the results from Approach 1, we found that Vp and Vc were both higher in the combination versus single administration groups. The covariate-adjusted popPK model fits the data well (Figures S6 and S7). However, as mentioned previously, the coefficients of these covariates cannot be directly interpreted as the isolated effect of coadministration. In addition, there was not a direct way to model the influence of coadministration on the levels of concentrations at various (unobserved) time-points between the two administrations. Hence, we consider these results based on Approach 2 as supportive.

## Discussion

To our knowledge, this is the first systematic analysis that examines the differences in PK of an HIV-1 mAb administered alone versus in combination with other HIV mAbs in the context of HIV prevention. Our analysis of data collected from three human clinical studies showed that the overall concentrations of VRC07-523LS over time did not differ significantly when it was given alone vs. combined with other mAbs. Specifically, although predicted concentrations of VRC07-523LS were slightly lower with combination administration at 1 day and 4 weeks post intravenous administration, they were not different between the two groups at 8- or 16-weeks post administration and beyond. The differences observed prior to week 4 are likely not clinically relevant in the context of HIV prevention because the mAb concentrations are sufficiently high for protection in both administration groups, though this requires further clinical investigation. On the other hand, the lack of differences in concentrations after week 8 is important because lower concentrations are more likely to contribute to differences in protection against HIV acquisition. Our results suggest that VRC07-523LS concentrations will remain essentially unaffected whether VRC07-523LS is administered alone or in combination with other mAbs, an important finding that supports coadministration of VRC07-523LS with other mAbs for HIV prevention. These findings also support the practicality of predicting VRC07-523LS serum concentrations based on PK models of data from regimens with the mAb being administered alone or in combination with other mAbs for the design of future combination mAb trials.

In addition to comparable overall mAb exposure, several PK parameters including clearance rate (CL), inter-compartmental clearance (Q), distribution half-life and overall exposure (AUC) did not differ significantly between the two administration groups. This finding aligns with those from another phase 1b/2a (‘CAPRISA 012A’) trial that observed comparable PK of VRC07-523LS alone and in combination [32]. However, no modeling was performed in CAPRISA 012A to formally investigate this question. We also confirmed earlier findings in these trials that VRC07-523LS concentrations increased with the administered doses, as expected, and that the dose of VRC07-523LS was a more important contributor to serum concentrations than whether the mAb was part of a combination or a single administration [40, 53, 62]. These data further support the linear pharmacokinetics modeling assumption. We also found that the mean covariate-adjusted Vc and Vp were slightly larger for combination versus single administration. This finding seems intuitive because the apparent volume required to distribute a greater amount of mAbs would be higher than for lower amount of mAbs. Because of the relationship between volume and half-lives, the time required to clear a greater number of antibodies would be consequently longer compared to eliminating a lower number of antibodies, as reflected in the slightly longer elimination half-life for the combination administration of VRC07-523LS.

Additionally, the estimated elimination half-lives (53 days for combination and 48 days for single) were longer than those reported in CAPRISA 012A for participants assigned female at birth in South Africa (29 days, combination administration) and VRC 605 in the US, the first-in-human trial of this mAb, (38 days for the intravenous route and 33 days for the subcutaneous route, single administration) [32, 44], but shorter than those found in the CAPRISA 012B study of participants assigned female at birth in South Africa (66 days, combination administration) [33]. These differences remain smaller than those between estimates from different studies when restricted to the same type of administration [32–34]. The difference in the elimination half-life in our study and the other studies could be due to differences in study population (mostly male and female USA participants vs South African women respectively), sample collection timepoints, assays for measuring serum concentrations, and PK analysis. In addition, the use of ENHANZE Drug Product (EDP), a recombinant human hyaluronidase designed to enhance absorption and, thus, PK profiles of subcutaneously administered drugs in CAPRISA 012B, could create heterogeneity [32, 33]. Importantly, the estimates from our analysis adjusted for participant characteristics for the purpose of comparing between the two administration groups. To our knowledge, this was not done in other studies. Taken together, the observed PK variability across studies demonstrates a need for continued research in this area, particularly since the elimination half-life is a critical variable for understanding the dosing parameters of these products for both prevention and treatment.

Our study has several notable strengths. First, we leveraged the larger sample size and the availability of data from both single and combination administrations from three studies that employed similar inclusion and exclusion criteria. This minimized any potential differences associated with study populations across studies while including more data points; this investigation would not be possible with any study alone. Second, we employed two different analytical approaches to compare VRC07-523LS PK and produced consistent findings. In addition, the results based on Approach 1 have an attractive interpretation in the causal framework, which is particularly important for comparisons between non-randomized groups. Lastly, serum measurements were performed with one single PK assay at a central HVTN laboratory for most of the specimens. This PK assay was optimized and validated, demonstrating excellent concordance of the observed concentrations with the true (known) concentration in serum, as well as precision, robustness and limits of detection and quantitation [76]. This was also one of the main reasons for not including data from other studies that used different PK assays, limiting technical (non-biological) variability in the underlying data due to measurement methods.

Our study did have limitations. We did not include data from other studies of VRC07-523LS, including VRC605, CAPRISA 012A and CAPRISA 012B, which would have further increased our sample size, as these studies had VRC07-523LS administered alone or combined with PGT121 or CAP256, respectively. However, as mentioned above, the benefit of including the additional studies does not outweigh the potential risks in sacrificing clarity in the interpretation of findings entailed by including data from different study populations, different sample collection time points, different laboratories, and different PK assays [32, 33]. Additionally, this study exclusively focuses on serum antibody levels but does not investigate neutralizing activity mediated by VRC07-523LS (which could possibly be analyzed with VRC07-523LS-only sensitive viruses). Participants in the studies we analyzed were mostly from the US with a small number from Switzerland. Future work is needed to incorporate data from ongoing studies to validate the findings in geographically diverse study populations. Future work is also needed to assess the impact of coadministration on safety, tolerability, or neutralization activity of VRC-07-523LS, which is outside the scope of this work.

Future prevention efficacy trials will employ dual or triple combinations with complementary regions of envelope glycoprotein targeted, to maximize viral coverage and reduce concerns about viral resistance. To our knowledge, our study is the first to report a comparison of the PK of an HIV-1 mAb between combination versus single administration for HIV-prevention in a pooled hypothesis-driven analysis systematically. It is reassuring that PK of VRC07-523LS appears to be altered only minimally when given with other anti-HIV antibodies.

## Supplementary Information

Supplementary Material 1. Supplemental materials contain additional tables and figures from both Approaches 1 and 2. Table S1: Summary of individual-level PK parameter estimates of VRC07-523LS from the base population PK model without covariate adjustment. Table S2: Estimated pharmacokinetic parameters of VRC07-523LS from a population PK model adjusted for coadministration. Figure S1: Observed VRC07-523LS serum concentrations over time. Figure S2: Observed individual-level VRC07-523LS serum concentrations over time for intravenous SPA and subcutaneous SPA. Figure S3: Observed individual-level VRC07-523LS serum concentrations over time for combination SPA and single SPA. Figure S4: Observed and predicted individual-level VRC07-523LS concentrations from the base popPK model of data pooled from the single and combination administration groups. Figure S5: Distributions of estimated individual-level PK features from the base popPK model prior to covariate adjustment. Figure S6: Observed and predicted individual-level VRC07-523LS serum concentrations from the covariate-adjusted popPK model of data pooled from the single and combination administration groups. Figure S7: Observed VRC07-523LS serum concentrations over time with the 90% prediction interval from the covariate-adjusted popPK model of data pooled from the single and combination administration groups.

## Data Availability

The data from HVTN 127/HPTN 087 and HVTN 130/HPTN 089 underlying the findings of this manuscript are publicly available at the HVTN website: https://atlas.scharp.org/cpas/project/HVTN%20Public%20Data/begin.view. The HVTN 136/HPTN 092 data will be available on the same website upon publication of the primary manuscript.

## References

[1] UNAIDS. UNAIDS Data 2023. 2023. https://www.unaids.org/en/resources/documents/2023/unaids-data.

[2] Karuna ST, Corey L. Broadly Neutralizing Antibodies for HIV Prevention. Annu Rev Med. 2020;71:329–46. 31986089 10.1146/annurev-med-110118-045506

[3] Prudden H, Tatoud R, Slack C, et al. Experimental medicine for HIV vaccine research and development. Vaccines (Basel). 2023;11:970. 37243074 10.3390/vaccines11050970 PMC10222747

[4] UNAIDS. Global HIV & AIDS Statistics - Fact Sheet 2022. Geneva, Switzerland. 2022.

[5] UNICEF. HIV treatment, care, and support for adolescents living with HIV in Eastern and Southern Africa: A review of interventions for scale. 2021. 2021.

[6] Van de Perre P, Scarlatti G, Moore PL, et al. Preventing breast milk HIV transmission using broadly neutralizing monoclonal antibodies: one size does not fit all. Immun Inflamm Dis. 2024;12:e1216. 38533917 10.1002/iid3.1216 PMC10966915

[7] Celum CL, Delany-Moretlwe S, Baeten JM, et al. HIV pre-exposure prophylaxis for adolescent girls and young women in Africa: from efficacy trials to delivery. J Int AIDS Soc. 2019;22(Suppl 4):e25298. 31328444 10.1002/jia2.25298 PMC6643076

[8] Delany-Moretlwe S, Hughes JP, Bock P, et al. Cabotegravir for the prevention of HIV-1 in women: results from HPTN 084, a phase 3, randomised clinical trial. Lancet. 2022;399:1779–89. 35378077 10.1016/S0140-6736(22)00538-4 PMC9077443

[9] Velloza J, Khoza N, Scorgie F, et al. The influence of HIV-related stigma on PrEP disclosure and adherence among adolescent girls and young women in HPTN 082: a qualitative study. J Int AIDS Soc. 2020;23:e25463. 32144874 10.1002/jia2.25463 PMC7060297

[10] World Health Organisation. Policy brief: pre-exposure prophylaxis (PrEP): WHO expands recommendation on oral pre-exposure prophylaxis of HIV infection (PrEP). World Health Organisation. 2015.

[11] Landovitz RJ, Donnell D, Clement ME, et al. Cabotegravir for HIV prevention in cisgender men and transgender women. N Engl J Med. 2021;385:595–608. 34379922 10.1056/NEJMoa2101016 PMC8448593

[12] Landovitz RJ, Hanscom BS, Clement ME, et al. Efficacy and safety of long-acting cabotegravir compared with daily oral tenofovir disoproxil fumarate plus emtricitabine to prevent HIV infection in cisgender men and transgender women who have sex with men 1 year after study unblinding: a secondary analysis of the phase 2b and 3 HPTN 083 randomised controlled trial. Lancet HIV. 2023;10:e767–78. 37952550 10.1016/S2352-3018(23)00261-8 PMC11375758

[13] Lowenthal ED, Chapman J, Ohrenschall R, et al. Acceptability and tolerability of long-acting injectable cabotegravir or rilpivirine in the first cohort of virologically suppressed adolescents living with HIV (IMPAACT 2017/MOCHA): a secondary analysis of a phase 1/2, multicentre, open-label, non-comparative dose-finding study. Lancet HIV. 2024;11:e222–32. 38538161 10.1016/S2352-3018(23)00301-6 PMC11061207

[14] Marzinke MA, Grinsztejn B, Fogel JM, et al. Characterization of Human Immunodeficiency Virus (HIV) infection in cisgender men and transgender women who Have sex with men receiving injectable cabotegravir for HIV prevention: HPTN 083. J Infect Dis. 2021;224:1581–92. 33740057 10.1093/infdis/jiab152 PMC8599849

[15] Marzinke MA, Hanscom B, Wang Z, et al. Efficacy, safety, tolerability, and pharmacokinetics of long-acting injectable cabotegravir for HIV pre-exposure prophylaxis in transgender women: a secondary analysis of the HPTN 083 trial. Lancet HIV. 2023;10:e703–12. 37783219 10.1016/S2352-3018(23)00200-X PMC10842527

[16] Murray MI, Markowitz M, Frank I, et al. Satisfaction and acceptability of cabotegravir long-acting injectable suspension for prevention of HIV: Patient perspectives from the ECLAIR trial. HIV Clin Trials. 2018;19:129–38. 30445896 10.1080/15284336.2018.1511346

[17] Tolley EE, Zangeneh SZ, Chau G, et al. Acceptability of long-acting injectable cabotegravir (CAB LA) in HIV-uninfected individuals: HPTN 077. AIDS Behav. 2020;24:2520–31. 32052214 10.1007/s10461-020-02808-2 PMC7423859

[18] Gilead. Press release: Gilead's twice-yearly Lenacapavir demonstrated 100% efficacy and superiority to daily Truvada for HIV prevention. 2024. https://www.gilead.com/news-and-press/press-room/press-releases/2024/6/gileads-twiceyearly-lenacapavir-demonstrated-100-efficacy-and-superiority-to-daily-truvada-for-hiv-prevention.

[19] Gray GE, Bekker LG, Laher F, et al. Vaccine efficacy of ALVAC-HIV and bivalent subtype C gp120-MF59 in Adults. N Engl J Med. 2021;384:1089–100. 33761206 10.1056/NEJMoa2031499 PMC7888373

[20] Gray GE, Mngadi K, Lavreys L et al. Phase llb efficacy trial of mosaic HIV-1 vaccine regimen in African women: Imbokodo. Conference on Retroviruses and Opportunistic Infections. Abstract 121; 2022: 2022.

[21] PrEPVacc. Press release: The vaccine trial in PrEPVacc. prepvacc.org/vacc. 2023. https://www.prepvacc.org/news/hiv-vaccines-tested-in-prepvacc-fail-to-reduce-infections-23-july-news-release*.*

[22] Aleshnick M, Florez-Cuadros M, Martinson T, Wilder BK. Monoclonal antibodies for malaria prevention. Mol Ther. 2022;30:1810–21. 35395399 10.1016/j.ymthe.2022.04.001 PMC8979832

[23] Bittner B, Richter W, Schmidt J. Subcutaneous administration of biotherapeutics: an overview of current challenges and opportunities. BioDrugs. 2018;32:425–40. 30043229 10.1007/s40259-018-0295-0 PMC6182494

[24] De KJ, Bakker AB, Marissen WE, et al. A human monoclonal antibody cocktail as a novel component of rabies postexposure prophylaxis. Annu Rev Med. 2007;58:359–68. 16886905 10.1146/annurev.med.58.061705.145053

[25] Deeks ED. Casirivimab/Imdevimab: First Approval. Drugs. 2021;81:2047–55. 34716907 10.1007/s40265-021-01620-z PMC8556815

[26] Fan Y, Lou J, Tam CC, et al. A three-monoclonal antibody combination potently neutralizes BoNT/G toxin in mice. Toxins (Basel). 2023;15:316. 37235351 10.3390/toxins15050316 PMC10222606

[27] Focosi D, Casadevall A. A critical analysis of the use of cilgavimab plus tixagevimab monoclonal antibody cocktail (Evusheld) for COVID-19 prophylaxis and treatment. Viruses. 2022;14:1999. 36146805 10.3390/v14091999 PMC9505619

[28] Howell KA, Brannan JM, Bryan C, et al. Cooperativity enables non-neutralizing antibodies to neutralize ebolavirus. Cell Rep. 2017;19:413–24. 28402862 10.1016/j.celrep.2017.03.049 PMC6082427

[29] Imbs DC, Negrier S, Cassier P, et al. Pharmacokinetics of pazopanib administered in combination with bevacizumab. Cancer Chemother Pharmacol. 2014;73:1189–96. 24705975 10.1007/s00280-014-2455-3

[30] Leonard JP, Friedberg JW, Younes A, et al. A phase I/II study of galiximab (an anti-CD80 monoclonal antibody) in combination with rituximab for relapsed or refractory, follicular lymphoma. Ann Oncol. 2007;18:1216–23. 17470451 10.1093/annonc/mdm114

[31] Liu X, Lu Y, Qin S. Atezolizumab and bevacizumab for hepatocellular carcinoma: mechanism, pharmacokinetics and future treatment strategies. Future Oncol. 2021;17:2243–56. 33663220 10.2217/fon-2020-1290

[32] Mahomed S, Garrett N, Capparelli EV, et al. Safety and pharmacokinetics of monoclonal antibodies VRC07-523LS and PGT121 administered subcutaneously for human immunodeficiency virus prevention. J Infect Dis. 2022;226:510–20. 35134995 10.1093/infdis/jiac041 PMC9417124

[33] Mahomed S, Garrett N, Capparelli EV, et al. Safety and pharmacokinetics of escalating doses of neutralising monoclonal antibody CAP256V2LS administered with and without VRC07-523LS in HIV-negative women in South Africa (CAPRISA 012B): a phase 1, dose-escalation, randomised controlled trial. Lancet HIV. 2023;10:e230–43. 37001964 10.1016/S2352-3018(23)00003-6

[34] Mayer BT, deCamp AC, Huang Y, et al. Optimizing clinical dosing of combination broadly neutralizing antibodies for HIV prevention. PLoS Comput Biol. 2022;18:e1010003. 35385469 10.1371/journal.pcbi.1010003 PMC9084525

[35] Ribrag V, Lee ST, Rizzieri D, et al. A phase 1b study to evaluate the safety and efficacy of durvalumab in combination with tremelimumab or danvatirsen in patients with relapsed or refractory diffuse large B-cell lymphoma. Clin Lymphoma Myeloma Leuk. 2021;21:309–17. 33632668 10.1016/j.clml.2020.12.012

[36] Stevenson L, Zinnack K, Donley J, Beebe L, Amaravadi L. Paradigm of combination biologics: analytical challenges related to pharmacokinetic assays and interpretation of pharmacokinetic and immunogenicity results. Bioanalysis. 2011;3:487–98. 21388262 10.4155/bio.10.214

[37] Yao M, Smart C, Hu Q, Cheng N. Continuous delivery of neutralising antibodies elevate CCL2 levels in mice bearing MCF10CA1d breast tumor xenografts. Transl Oncol. 2017;10:734–43. 28734227 10.1016/j.tranon.2017.06.009 PMC5521028

[38] Unitaid, IAVI, Wellcome, Medicines Patent Pool. Novel business models for accessible monoclonal antibodies for infectious diseases in low- and middle-income countries: Recommendations from a multistakeholder meeting convened by IAVI, Unitaid, the Medicines Patent Pool, and Wellcome. 2023.

[39] Corey L, Gilbert PB, Juraska M, et al. Two randomized trials of neutralizing antibodies to prevent HIV-1 acquisition. N Engl J Med. 2021;384:1003–14. 33730454 10.1056/NEJMoa2031738 PMC8189692

[40] Sobieszczyk ME, Mannheimer S, Paez CA, et al. Safety, tolerability, pharmacokinetics, and immunological activity of dual-combinations and triple-combinations of anti-HIV monoclonal antibodies PGT121, PGDM1400, 10–1074, and VRC07-523LS administered intravenously to HIV-uninfected adults: a phase 1 randomised trial. Lancet HIV. 2023;10:e653–62. 37802566 10.1016/S2352-3018(23)00140-6 PMC10629933

[41] Seaton KE, Huang Y, Karuna S, et al. Pharmacokinetic serum concentrations of VRC01 correlate with prevention of HIV-1 acquisition. EBioMedicine. 2023;93:104590. 37300931 10.1016/j.ebiom.2023.104590 PMC10363420

[42] Reeves DB, Mayer BT, deCamp AC, et al. High monoclonal neutralization titers reduced breakthrough HIV-1 viral loads in the antibody mediated prevention trials. Nat Commun. 2023;14:8299. 38097552 10.1038/s41467-023-43384-y PMC10721814

[43] Mgodi NM, Takuva S, Edupuganti S, et al. A Phase 2b study to evaluate the safety and efficacy of VRC01 broadly neutralizing monoclonal antibody in reducing acquisition of HIV-1 infection in women in Sub-Saharan Africa: baseline findings. J Acquir Immune Defic Syndr. 2021;87:680–7. 33587510 10.1097/QAI.0000000000002649 PMC8436719

[44] Gaudinski MR, Houser KV, Doria-Rose NA, et al. Safety and pharmacokinetics of broadly neutralising human monoclonal antibody VRC07-523LS in healthy adults: a phase 1 dose-escalation clinical trial. Lancet HIV. 2019;6:e667–79. 31473167 10.1016/S2352-3018(19)30181-X PMC11100866

[45] Mayer KH, Seaton KE, Huang Y, et al. Safety, pharmacokinetics, and immunological activities of multiple intravenous or subcutaneous doses of an anti-HIV monoclonal antibody, VRC01, administered to HIV-uninfected adults: Results of a phase 1 randomized trial. PLoS Med. 2017;14:e1002435. 29136037 10.1371/journal.pmed.1002435 PMC5685476

[46] Takuva S, Karuna ST, Juraska M, et al. Infusion reactions after receiving the broadly neutralizing antibody VRC01 or placebo to reduce HIV-1 acquisition: results from the phase 2b antibody-mediated prevention randomized trials. J Acquir Immune Defic Syndr. 2022;89:405–13. 34923559 10.1097/QAI.0000000000002892 PMC9555144

[47] Cohen YZ, Butler AL, Millard K, et al. Safety, pharmacokinetics, and immunogenicity of the combination of the broadly neutralizing anti-HIV-1 antibodies 3BNC117 and 10–1074 in healthy adults: a randomized, phase 1 study. PLoS ONE. 2019;14:e0219142. 31393868 10.1371/journal.pone.0219142 PMC6687118

[48] Julg B, Stephenson KE, Wagh K, et al. Safety and antiviral activity of triple combination broadly neutralizing monoclonal antibody therapy against HIV-1: a phase 1 clinical trial. Nat Med. 2022;28:1288–96. 35551291 10.1038/s41591-022-01815-1 PMC9205771

[49] Pyzik M, Kozicky LK, Gandhi AK, Blumberg RS. The therapeutic age of the neonatal Fc receptor. Nat Rev Immunol. 2023;23:415–32. 36726033 10.1038/s41577-022-00821-1 PMC9891766

[50] Jin F, Balthasar JP. Mechanisms of intravenous immunoglobulin action in immune thrombocytopenic purpura. Hum Immunol. 2005;66:403–10. 15866704 10.1016/j.humimm.2005.01.029

[51] Jin F, Tayab ZR, Balthasar JP. Pharmacokinetic and pharmacodynamic effects of high-dose monoclonal antibody therapy in a rat model of immune thrombocytopenia. AAPS J. 2006;7:E895–902. 16594642 10.1208/aapsj070487 PMC2750959

[52] Walsh SR, Seaman MS. Broadly neutralizing antibodies for HIV-1 prevention. Front Immunol. 2021;12:712122. 34354713 10.3389/fimmu.2021.712122 PMC8329589

[53] Edupuganti S, Hurt CB, Stephenson KE et al. First-in-human evaluation of safety and pharmacokinetics of intravenous or subcutaneous infusions of PGT121.141.LS, an anti-V3 HIV-1 broadly neutralising in healthy adult volunteers without HIV. *AIDS 2022;* 2022.

[54] Walsh SR, Gay CL, Karuna ST, et al. Safety and pharmacokinetics of VRC07-523LS administered via different routes and doses (HVTN 127/HPTN 087): a Phase I randomized clinical trial. PLoS Med. 2024;21:e1004329. 38913710 10.1371/journal.pmed.1004329 PMC11251612

[55] Huang Y, Zhang L, Ledgerwood J, et al. Population pharmacokinetics analysis of VRC01, an HIV-1 broadly neutralizing monoclonal antibody, in healthy adults. MAbs. 2017;9:792–800. 28368743 10.1080/19420862.2017.1311435 PMC5524155

[56] Awan SF, Pegu A, Strom L, et al. Phase 1 trial evaluating safety and pharmacokinetics of HIV-1 broadly neutralizing mAbs 10E8VLS and VRC07–523LS. JCI Insight. 2024;9:e175375. 38587079 10.1172/jci.insight.175375 PMC11128198

[57] Gilbert PB, Huang Y, deCamp AC, et al. Neutralization titer biomarker for antibody-mediated prevention of HIV-1 acquisition. Nat Med. 2022;28:1924–32. 35995954 10.1038/s41591-022-01953-6 PMC9499869

[58] Mayer BT, Zhang L, deCamp AC, et al. Impact of LS mutation on pharmacokinetics of preventive HIV broadly neutralizing monoclonal antibodies: a cross-protocol analysis of 16 clinical trials in people without HIV. Pharmaceutics. 2024;16:594. 38794258 10.3390/pharmaceutics16050594 PMC11125931

[59] Miner MD, Corey L, Montefiori D. Broadly neutralizing monoclonal antibodies for HIV prevention. J Int AIDS Soc. 2021;24(Suppl 7):e25829. 34806308 10.1002/jia2.25829 PMC8606861

[60] Mkhize NN, Yssel AEJ, Kaldine H, et al. Neutralization profiles of HIV-1 viruses from the VRC01 Antibody Mediated Prevention (AMP) trials. PLoS Pathog. 2023;19:e1011469. 37384759 10.1371/journal.ppat.1011469 PMC10337935

[61] Rudicell RS, Kwon YD, Ko SY, et al. Enhanced potency of a broadly neutralizing HIV-1 antibody in vitro improves protection against lentiviral infection in vivo. J Virol. 2014;88:12669–82. 25142607 10.1128/JVI.02213-14 PMC4248941

[62] Walsh SR, Gay CL, Karuna ST et al. A Randomised Clinical Trial of the Safety and Pharmacokinetics of VRC07–523LS Administered via Different Routes and Doses (HVTN 127/HPTN 087). *medRxiv* 2024. 10.1371/journal.pmed.1004329 PMC11251612 38913710

[63] Wesley MS, Chiong KT, Seaton KE, et al. Validation of a triplex pharmacokinetic assay for simultaneous quantitation of HIV-1 broadly neutralizing antibodies PGT121, PGDM1400, and VRC07-523-LS. Front Immunol. 2021;12:709994. 34504492 10.3389/fimmu.2021.709994 PMC8422903

[64] Chaffee PH, van der Laan MJ. Targeted maximum likelihood estimation for dynamic treatment regimes in sequentially randomized controlled trials. Int J Biostat. 2012;8:Article 14. 22740582 10.1515/1557-4679.1406 PMC6084784

[65] Lendle SD, Fireman B, van der Laan MJ. Targeted maximum likelihood estimation in safety analysis. J Clin Epidemiol. 2013;66:S91–8. 23849159 10.1016/j.jclinepi.2013.02.017 PMC3818128

[66] Rytgaard HCW, Eriksson F, van der Laan MJ. Estimation of time-specific intervention effects on continuously distributed time-to-event outcomes by targeted maximum likelihood estimation. Biometrics. 2023;79:3038–49. 36988158 10.1111/biom.13856

[67] Rytgaard HCW, van der Laan MJ. Targeted maximum likelihood estimation for causal inference in survival and competing risks analysis. Lifetime Data Anal. 2024;30:4–33. 36336732 10.1007/s10985-022-09576-2

[68] van der Laan MJ. Targeted maximum likelihood based causal inference: Part II. Int J Biostat. 2010;6(2):Article 3. 10.2202/1557-4679. 21731531 10.2202/1557-4679.1241 PMC3126672

[69] van der Laan MJ. Targeted maximum likelihood based causal inference: Part I. Int J Biostat. 2010;6(2):Article 2. 10.2202/1557-4679.1211. 21969992 10.2202/1557-4679.1211 PMC3126670

[70] *Monolix 2021R2, Lixoft SAS, a Simulations Plus company.* 2021.

[71] Ayral G, Si Abdallah JF, Magnard C, Chauvin J. A novel method based on unbiased correlations tests for covariate selection in nonlinear mixed effects models: The COSSAC approach. CPT Pharmacometrics Syst Pharmacol. 2021;10:318–29. 33755345 10.1002/psp4.12612 PMC8099437

[72] Huang Y, Naidoo L, Zhang L, et al. Pharmacokinetics and predicted neutralisation coverage of VRC01 in HIV-uninfected participants of the Antibody Mediated Prevention (AMP) trials. EBioMedicine. 2021;64:103203. 33493795 10.1016/j.ebiom.2020.103203 PMC7841500

[73] Cockcroft DW, Gault MH. Prediction of creatinine clearance from serum creatinine. Nephron. 1976;16:31–41. 1244564 10.1159/000180580

[74] R Core Team. R: A language and environment for statistical computing. R Foundation for statistical computing. Vienna, Austria; 2022. https://cran.r-project.org/doc/manuals/r-release/fullrefman.pdf.

[75] Holm S. A simple sequentially rejective multiple test procedure. Scand J Stat. 1979;6:65–70.

[76] Seaton KE, Huang Y, Karuna S, et al. Pharmacokinetic serum concentrations of VRC01 correlate with prevention of HIV-1 acquisition. EBioMedicine. 2023;93:104590. 37300931 10.1016/j.ebiom.2023.104590 PMC10363420

