# Supplementary material for: Pharmacokinetic interaction assessment of an HIV broadly neutralizing monoclonal antibody VRC07-523LS: a cross-protocol analysis of three phase 1 trials in people without HIV
Source: BMC Immunol. 2025 Feb 19;26:8. doi: 10.1186/s12865-025-00687-7 (PMC11837431; doi:10.1186/s12865-025-00687-7)
Supplement: Supplementary file 1 — Supplementary Material 1. Supplemental materials contain additional tables and figures from both Approaches 1 and 2. Table S1: Summary of individual-level PK parameter estimates of VRC07-523LS from the base population PK model without covariate adjustment. Table S2: Estimated pharmacokinetic parameters of VRC07-523LS from a population PK model adjusted for coadministration. Figure S1: Observed VRC07-523LS serum concentrations over time. Figure S2: Observed individual-level VRC07-523LS serum concentrations over time for intravenous SPA and subcutaneous SPA. Figure S3: Observed individual-level VRC07-523LS serum concentrations over time for combination SPA and single SPA. Figure S4: Observed and predicted individual-level VRC07-523LS concentrations from the base popPK model of data pooled from the single and combination administration groups. Figure S5: Distributions of estimated individual-level PK features from the base popPK model prior to covariate adjustment. Figure S6: Observed and predicted individual-level VRC07-523LS serum concentrations from the covariate-adjusted popPK model of data pooled from the single and combination administration groups. Figure S7: Observed VRC07-523LS serum concentrations over time with the 90% prediction interval from the covariate-adjusted popPK model of data pooled from the single and combination administration groups. [file 12865_2025_687_MOESM1_ESM.docx]

**Supplemental Materials**

**Table S1: Summary of individual-level PK parameter estimates of VRC07-523LS from the base population PK model without covariate adjustment.** *Abbreviations: CL- clearance rate; Vc- central volume; Q- inter-compartmental clearance; Vp- peripheral volume; C_maxADJ_: dose- and weight-adjusted maximum concentration; AUC_ADJ_- dose- and weight-adjusted area under the curve; SD – standard deviation.*

| **Parameter** | **Combination administration (N=46)** | | | | | **Single administration (N=100)** | | | | |
| --- | --- | --- | --- | --- | --- | --- | --- | --- | --- | --- |
|  | **Median** | **Min** | **Max** | **Mean** | **SD** | **Median** | **Min** | **Max** | **Mean** | **SD** |
| **CL (L/day)** | 0.13 | 0.06 | 0.21 | 0.13 | 0.03 | 0.12 | 0.06 | 0.29 | 0.12 | 0.04 |
| **Vc (L)** | 4.00 | 1.67 | 9.81 | 4.60 | 1.69 | 3.49 | 1.76 | 10.10 | 3.93 | 1.54 |
| **Q (L/day)** | 0.31 | 0.09 | 0.69 | 0.33 | 0.12 | 0.31 | 0.11 | 0.59 | 0.31 | 0.10 |
| **Vp (L)** | 3.88 | 2.31 | 5.85 | 3.89 | 0.76 | 3.57 | 2.62 | 4.67 | 3.54 | 0.45 |

**Table S2: Estimated pharmacokinetic (PK) parameters of VRC07-523LS from a population PK model adjusted for coadministration (Combination vs. Single) (Approach 2).** *Abbreviations: Coadmn- coadministration; CI- confidence interval; %RSE - % relative standard error; SD: standard deviation; SE: standard error.*

| **Parameters** | **Description** | **Estimate** | **95% CI** | **%RSE** |
| --- | --- | --- | --- | --- |
| **Fixed Effects** | | | | |
| *F* | Bioavilability | 0.45 | (0.42, 0.48) | 3.60 |
| *k_a_* (/day) | Absorption rate constant | 0.32 | (0.28, 0.38) | 7.82 |
| *CL* (L/day) | Clearance | 0.06 | (0.04, 0.07) | 10.61 |
| *V_c_* (L) | Central volume | 3.21 | (2.89, 3.56) | 5.29 |
| *Q* (L/day) | Inter-compartmental clearance | 0.09 | (0.05, 0.17) | 35.98 |
| *V_p_* (L) | Peripheral volume | 1.24 | (0.92, 1.66) | 15.13 |
| $\beta_{VpCoadm}$ | Adjusted fixed effect of peripheral volume for coadministration | 0.12 | (0.06, 0.24) | 40.88 |
| $\beta_{VcCoadm}$ | Adjusted fixed effect of central volume for coadministration | 0.23 | (0.13, 0.41) | 32.62 |
| $\beta_{Clweight}$ | Adjusted fixed effect of clearance for weight | 0.01 | (0.01, 0.01) | 13.58 |
| $\beta_{Qweight}$ | Adjusted fixed effect of inter-compartmental clearance for weight | 0.02 | (0.01, 0.03) | 23.64 |
| $\beta_{Vpweight}$ | Adjusted fixed effect of peripheral volume for weight | 0.01 | (0.01, 0.02) | 11.90 |
| **Random Effects** | | | | |
| $\omega_{CL}$ | SD, clearance | 0.25 | (0.22, 0.28) | 6.82 |
| $\omega_{Vc}$ | SD, central volume | 0.33 | (0.28, 0.39) | 8.62 |
| $\omega_{Q}$ | SD, inter-compartmental clearance | 0.56 | (0.46, 0.69) | 10.70 |
| $\omega_{Vp}$ | SD, peripheral volume | 0.22 | (0.18, 0.26) | 10.36 |
| **Correlations** | | | | |
| $\rho_{Vp Cl}$ | Correlation between random effects for *V_p_* and *CL* | 0.82 | (0.68, 0.90) | 7.11 |
| **Error Model Parameters** | | | | |
| $\sigma(constant)$ | SE, additive | 0.16 | (0.12, 0.23) | 17.71 |
| $\sigma(proportional)$ | SE, proportional | 0.14 | (0.13, 0.15) | 3.38 |


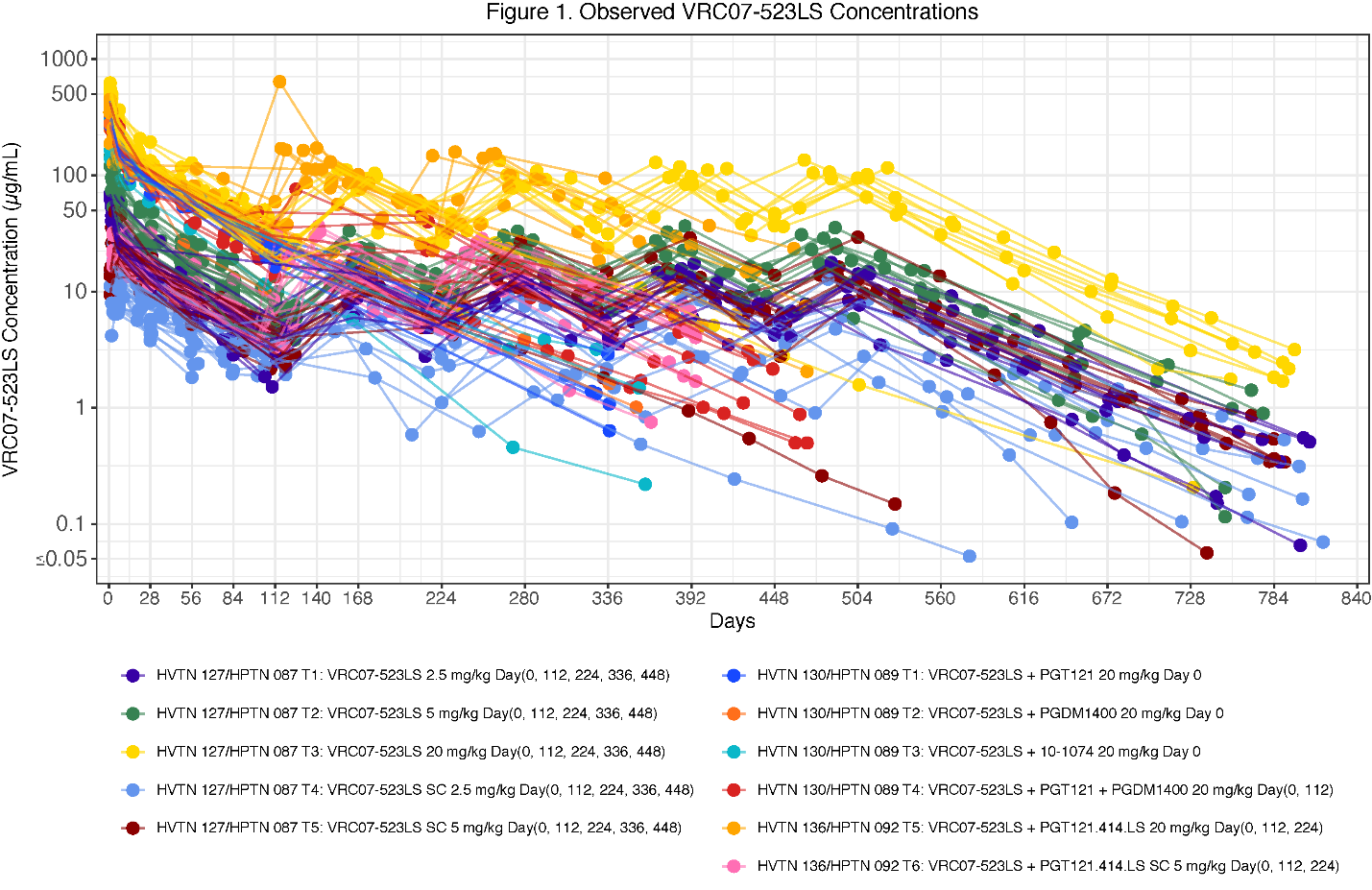
**Figure S1: Observed VRC07-523LS serum concentrations over time.** Different colors of the points indicate data from different dosing regimens in the three phase 1 trials. This figure shows the same data as Figure 2, but with a different coloring scheme. Each line in the graph indicates a unique participant. Each dot in the graph indicates an observed concentration data point for a given participant.

| **A** |
| --- |
| 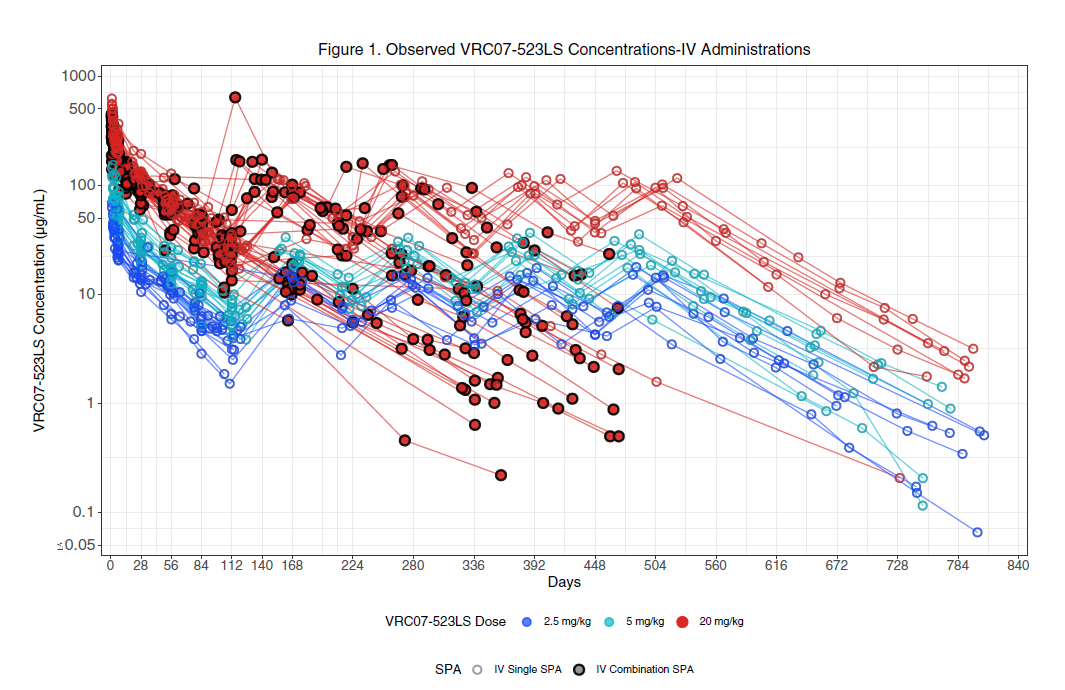 |
| **B** |
| **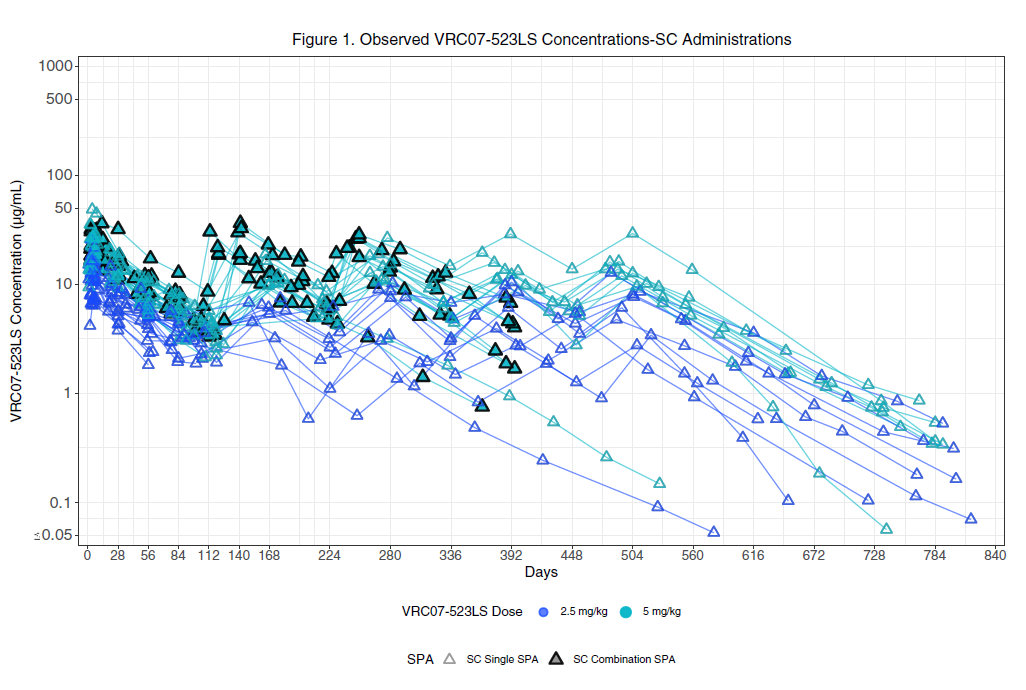** |

**Figure S2: Observed individual-level VRC07-523LS serum concentrations over time for intravenous SPA (Panel A) and subcutaneous SPA (Panel B).** Colors represent different dose levels of VRC07-523LS at 2.5, 5 or 20 mg/kg; shapes represent route of administration via subcutaneous or intravenous; filled vs. open symbols represent combination vs. single administrations, respectively. Collectively, this figure also shows the same data as Figure 2, separating the IV and SC groups in two separate panels. Each line in the graph indicates a unique participant. Each symbol in the graph indicates an observed concentration data point for a given participant. *Abbreviations:* *IV- intravenous; SC- subcutaneous;* *SPA: study product administration.*

| **A** |
| --- |
| 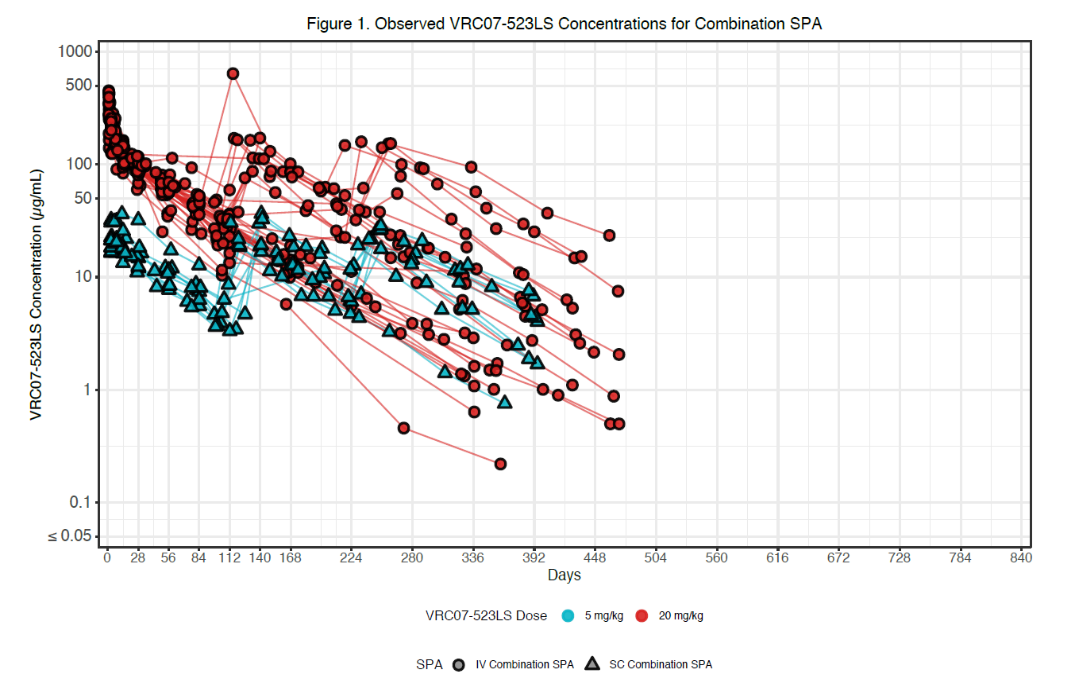 |
| **B** |
| **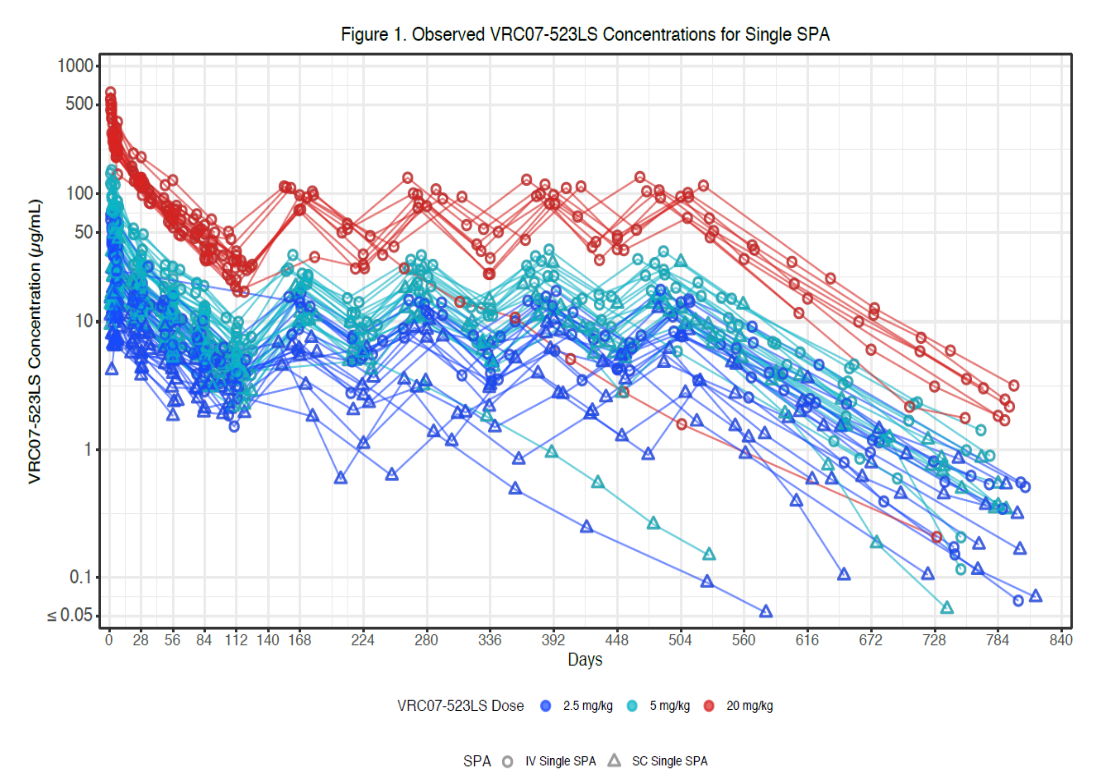** |

**Figure S3: Observed individual-level VRC07-523LS serum concentrations over time for combination SPA (Panel A) and single SPA (Panel B).** Colors represent different dose levels of VRC07-523LS at 2.5, 5 or 20 mg/kg; shapes represent route of administration via subcutaneous or intravenous; filled vs. open symbols represent combination vs. single administrations, respectively. Collectively, this figure also shows the same data as Figure 2, separating the combination and single groups in two separate panels. Each line in the graph indicates a unique participant. Each line in the graph indicates a unique participant. Each symbol in the graph indicates an observed concentration data point for a given participant. *Abbreviations:* *IV- intravenous; SC- subcutaneous;* *SPA: study product administration.*


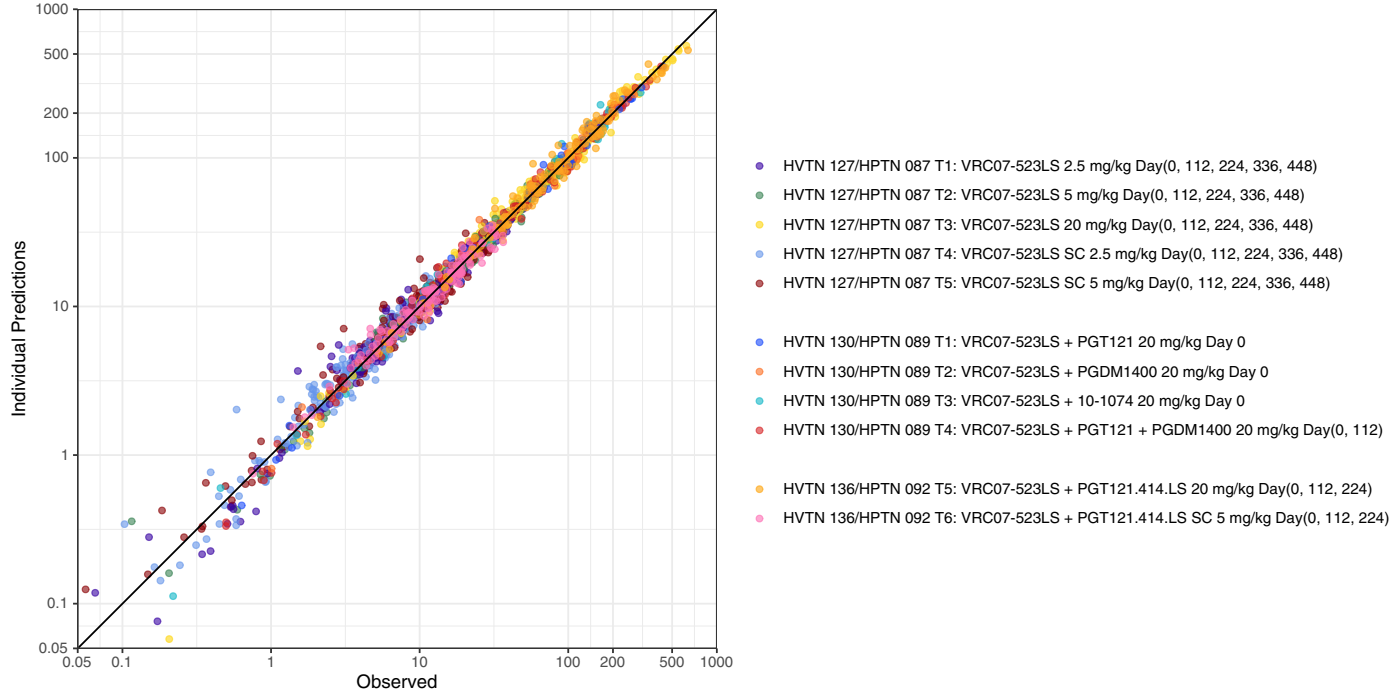


Figure S4: Observed and predicted individual-level VRC07-523LS serum concentrations from the base popPK model of data pooled from the single and combination administration groups. Different colors of the points indicate data from different dosing regimens in the three phase 1 trials.


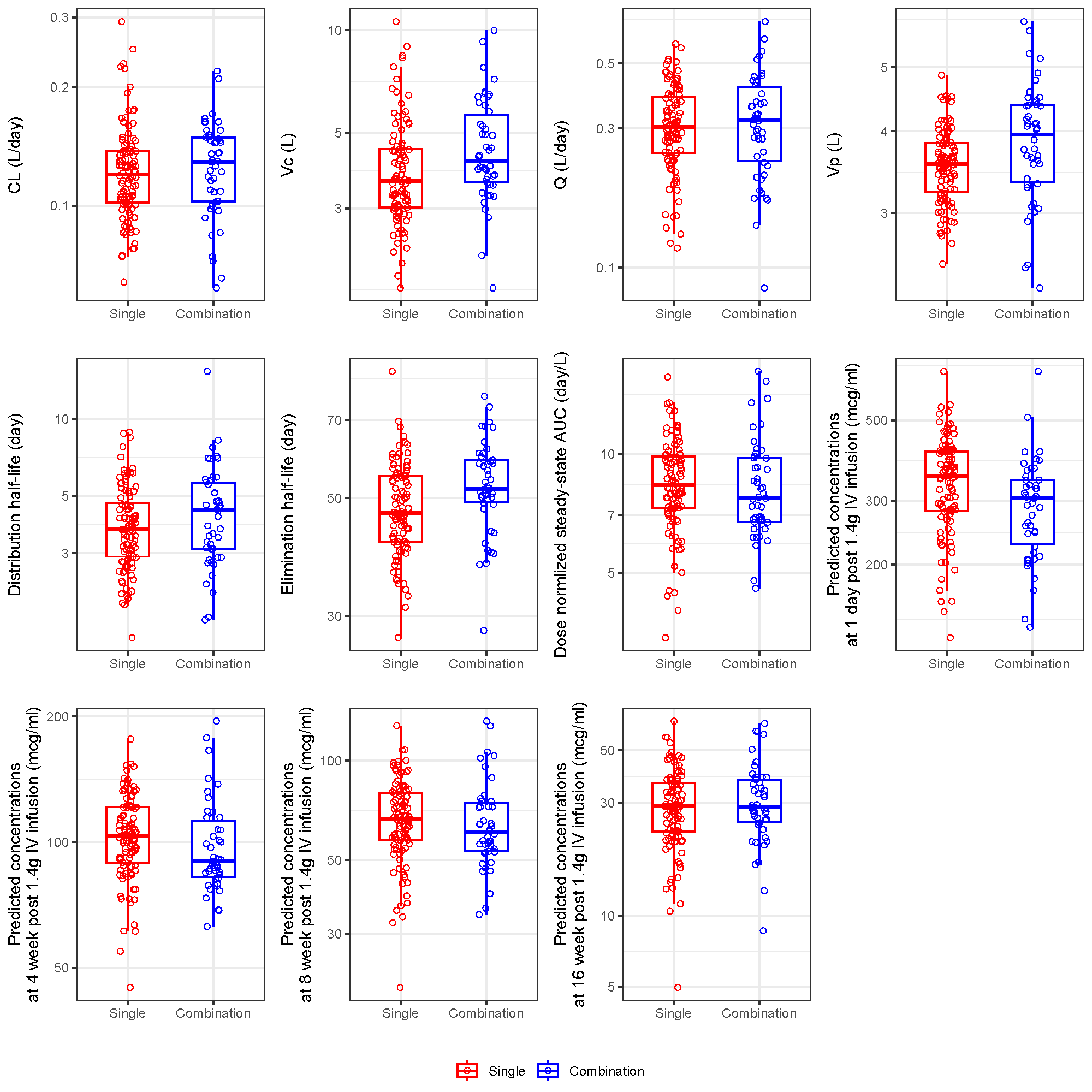


Figure S5: Distributions of estimated individual-level PK parameters and features derived from the base popPK model prior to covariate adjustment. *Abbreviations: CL- clearance rate; Vc- central volume; Q- inter-compartmental clearance; Vp- peripheral volume; AUC- area under the curve; IV- intravenous.*


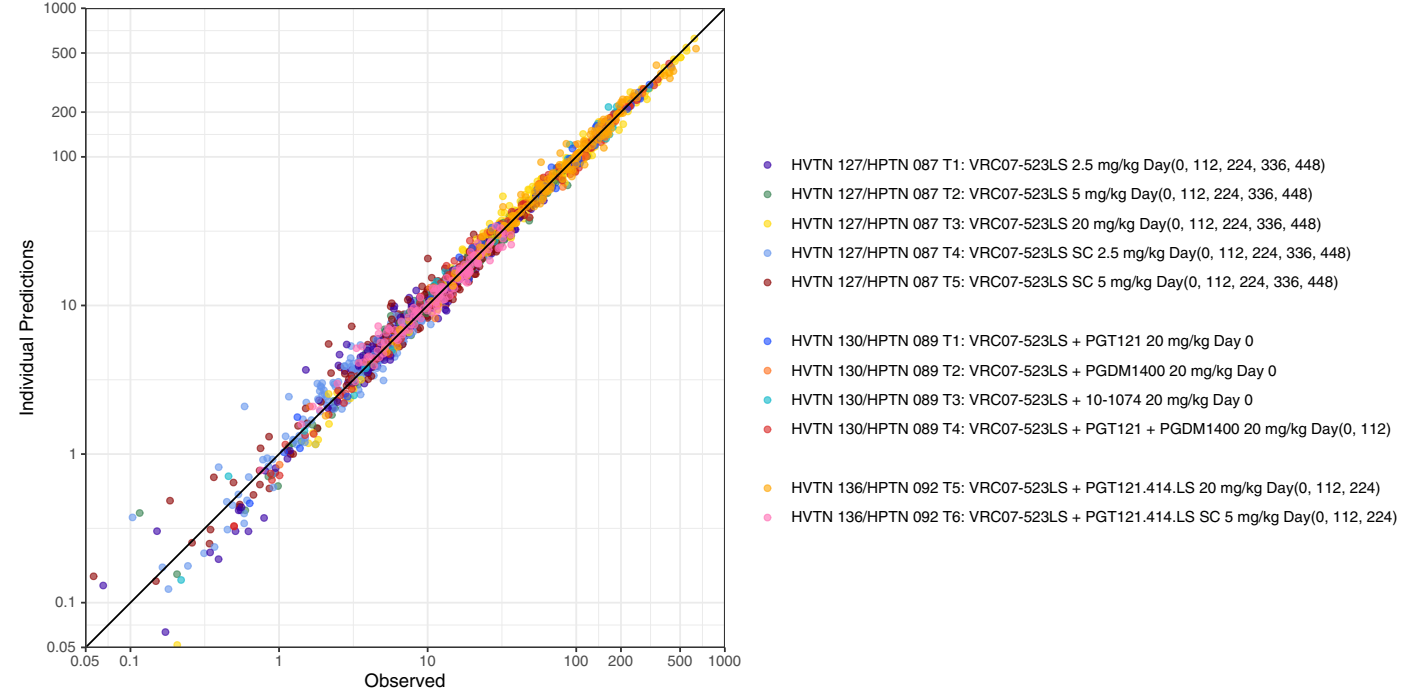


Figure S6: Observed and predicted individual-level VRC07-523LS serum concentrations from the covariate-adjusted popPK model of data pooled from the single and combination administration groups. Coadministration (combination vs. single) and body weight were included as covariates in the model as shown in Table S2. Different colors of the points indicate data from different dosing regimens in the three phase 1 trials.


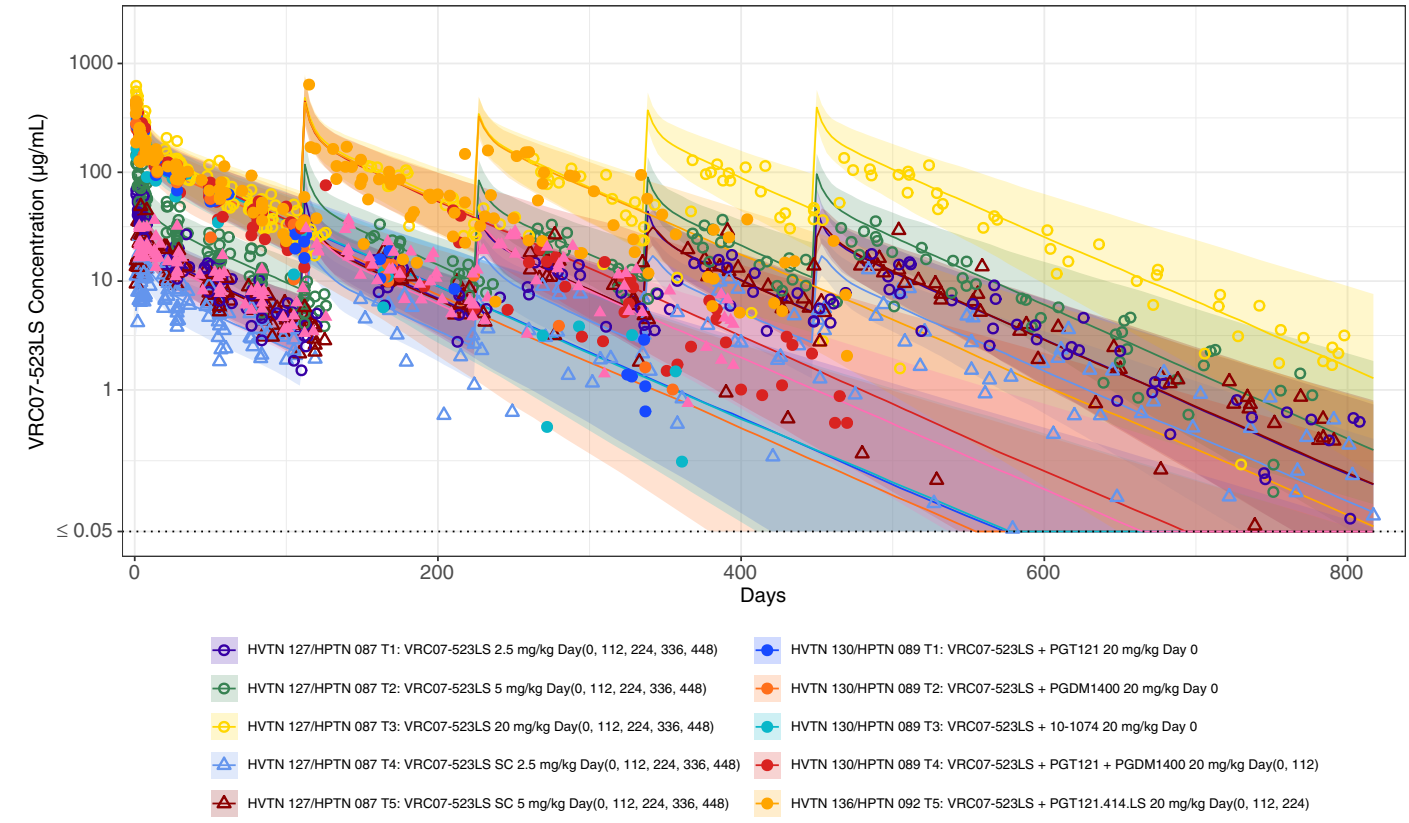


Figure S7: Observed VRC07-523LS serum concentrations over time with the 90% prediction interval from the covariate-adjusted popPK model of data pooled from the single and combination administration groups. Coadministration (combination vs. single) and body weight were included as covariates in the model as shown in Table S2. Symbols and colors represent the observed concentrations from different dosing regimens with circles indicating those after an IV administration and triangles indicating those after a SC administration. Solid lines represent the predicted median concentration of each study regimen, with shaded areas denoting the 90% prediction interval.
